# Genetic diversity, linkage disequilibrium and power of a large grapevine (*Vitis vinifera* L) diversity panel newly designed for association studies

**DOI:** 10.1186/s12870-016-0754-z

**Published:** 2016-03-22

**Authors:** Stéphane D. Nicolas, Jean-Pierre Péros, Thierry Lacombe, Amandine Launay, Marie-Christine Le Paslier, Aurélie Bérard, Brigitte Mangin, Sophie Valière, Frédéric Martins, Loïc Le Cunff, Valérie Laucou, Roberto Bacilieri, Alexis Dereeper, Philippe Chatelet, Patrice This, Agnès Doligez

**Affiliations:** INRA, UMR AGAP, F-34060 Montpellier, France; GQE-Le Moulon, INRA - Univ. Paris-Sud - CNRS - AgroParisTech - Université Paris-Saclay, Ferme du Moulon, F-91190 Gif-sur-Yvette, France; INRA, US1279 EPGV, CEA-IG/CNG, F-91057 Evry, France; INRA, UR MIAT, F-31326 Castanet-Tolosan, France; INRA, Plateforme Génomique, F-31326 Castanet-Tolosan, France; INSERM, UMR1048, F-31432 Toulouse, France; IFV, UMT Genovigne, F-34060 Montpellier, France; IRD, UMR IPME, F-34394 Montpellier 5, France

**Keywords:** *Vitis*, Association panel, Linkage disequilibrium, Power, Genome-wide association studies, SSR, SNP, *sylvestris*, Vassal collection, Haplotype, Kinship

## Abstract

**Background:**

As for many crops, new high-quality grapevine varieties requiring less pesticide and adapted to climate change are needed. In perennial species, breeding is a long process which can be speeded up by gaining knowledge about quantitative trait loci linked to agronomic traits variation. However, due to the long juvenile period of these species, establishing numerous highly recombinant populations for high resolution mapping is both costly and time-consuming. Genome wide association studies in germplasm panels is an alternative method of choice, since it allows identifying the main quantitative trait loci with high resolution by exploiting past recombination events between cultivars. Such studies require adequate panel design to represent most of the available genetic and phenotypic diversity. Assessing linkage disequilibrium extent and panel power is also needed to determine the marker density required for association studies.

**Results:**

Starting from the largest grapevine collection worldwide maintained in Vassal (France), we designed a diversity panel of 279 cultivars with limited relatedness, reflecting the low structuration in three genetic pools resulting from different uses (table *vs* wine) and geographical origin (East *vs* West), and including the major founders of modern cultivars. With 20 simple sequence repeat markers and five quantitative traits, we showed that our panel adequately captured most of the genetic and phenotypic diversity existing within the entire Vassal collection. To assess linkage disequilibrium extent and panel power, we genotyped single nucleotide polymorphisms: 372 over four genomic regions and 129 distributed over the whole genome. Linkage disequilibrium, measured by correlation corrected for kinship, reached 0.2 for a physical distance between 9 and 458 Kb depending on genetic pool and genomic region, with varying size of linkage disequilibrium blocks. This panel achieved reasonable power to detect associations between traits with high broad-sense heritability (> 0.7) and causal loci with intermediate allelic frequency and strong effect (explaining > 10 % of total variance).

**Conclusions:**

Our association panel constitutes a new, highly valuable resource for genetic association studies in grapevine, and deserves dissemination to diverse field and greenhouse trials to gain more insight into the genetic control of many agronomic traits and their interaction with the environment.

**Electronic supplementary material:**

The online version of this article (doi:10.1186/s12870-016-0754-z) contains supplementary material, which is available to authorized users.

## Background

Grape (*Vitis vinifera*) is a crop of major economic importance. Worldwide, 73.7 million tonnes of grapes were produced on 7.5 million ha in 2014, and wine trade represented a gross value of 25.6 billion euros [1]. This high value crop requires adaptation to upcoming climate changes [2]. According to the least optimistic predictions, most major wine producing regions could become by 2050 unsuitable for currently grown cultivars [3, 4]. In addition, viticulture is required to reduce pesticides use, grapevine being one of the most intensively treated crops. It is therefore crucial to rapidly breed new adapted and resistant cultivars. In this perennial species with a long juvenile period, breeding is still a slow process although knowledge of the genetic determinism of agronomic traits is just emerging to speed up breeding through marker assisted selection [5–9].

*V. vinifera* domestication began in the Near East 6000–8000 years ago [10, 11] and cultivars then found their way to most European, Northern African and Eastern countries through different routes. A large number of diverse cultivars (*V. vinifera* subsp. *vinifera*) are used for fruit and juice consumption (table grape) and/or wine production (wine grape). By contrast, a few relict populations of wild grapes (*V. vinifera* subsp. *sylvestris*) still occupy limited areas mainly in Mediterranean countries. The possible contribution of Western Europe wild populations to the development of present cultivars during the diffusion of grapevine is still debated [12, 13]. Diversity and patterns of population structure have recently been clarified for cultivated grapes using molecular data [12, 14–16]. These studies confirmed the three genetic pools previously established based on morphological traits [17]: Western wine, Eastern wine and Eastern table. In addition, deoxyribonucleic acid (DNA) polymorphisms have been very useful to refine this population structure through the identification of subgroups corresponding to specific geographical locations and ultimately to kinship groups [15]. Cultivars constitute a complex network involving many close pedigree relationships [14, 18], indicating that the available diversity has not been fully utilized for breeding purposes.

Compared to other crops such as corn or tomato, only a few quantitative trait loci (QTLs) have been detected in *V. vinifera*, each trait of interest being studied in a single or very few crosses. The genetic control of major agronomic traits such as fertility, phenology, berry weight, seedlessness, berry phenolic composition and adaptation to abiotic stresses has been partially elucidated (e.g. [19–28]). However, the wide diversity in cultivated grapevine remains largely underexplored.

Genome-wide association studies (GWAS) in germplasm samples are more efficient than family-based mapping for QTL detection in highly diverse perennial species, in which producing and phenotyping large bi- or multi-parental populations segregating for different agronomic traits is very time-consuming and costly [29]. Compared to QTL detection in such progenies, GWAS in panels of accessions is not limited to causal polymorphisms segregating in parents, and provides a higher mapping resolution [30]. GWAS indeed uses all past recombination events that occurred during the successive generations separating common ancestors from individuals in the study panel. GWAS power strongly depends on (i) linkage disequilibrium (LD) between causal polymorphisms and markers within the panel [31–33], (ii) factors related to panel design (size, genetic structure, relatedness), traits (heritability, genetic architecture) and causal loci (QTL effect, allelic frequency) [33, 34], (iii) statistical model used to detect associations [33, 35] and methods used to correct for multiple testing [36].

Since LD can largely vary across and within species depending on the individuals assembled in diversity panels [37], it is of utmost importance to estimate LD extent in panels before applying GWAS, in order to evaluate the density of molecular markers required to achieve a given power. Simulating the power of association tests in such panels is very useful to delineate the range of trait heritability, minor allelic frequency, locus differentiation and QTL effect yielding efficient association detection. Power simulation is also useful for choosing the best kinship estimator to maximize power without increasing false positive rate [33].

Linkage disequilibrium extent has previously been estimated in *V. vinifera*, for both simple sequence repeat (SSR) and single nucleotide polymorphism (SNP) markers. Barnaud et al [38, 39] reported significant LD values between SSRs extending to 14–17 centiMorgans (cM) in a core collection of cultivars and to less than 1 cM in a wild sample (1 cM corresponding on average in *V. vinifera* to about 300–400 Kb for a total genome size of 487–504.6 Mb [40–43]). By contrast, LD decays much more rapidly between SNPs, with *r*^2^ values reaching 0.2 within a few Kb at most [14, 44]. However, the variation in LD extent among genetic pools has not been explored in grapevine yet.

Several *V. vinifera* subsp. *vinifera* core collections have been defined by maximizing global diversity, based either on morphological [38] or genetic data [16, 45]. They have proved useful for efficient screening of diversity, since they capture most extreme phenotypes or rare alleles (e.g. [46]). They have also been used in association genetics to test a few candidate genes [21, 24, 47–49]. However, new genotyping technologies allow the development of association studies based on more relevant, larger-sized panels, representing more evenly the diversity from each of the three cultivated *V. vinifera* genetic pools. A genome-wide association study has already been applied to the United States Department of Agriculture (USDA) collection, which partially represents *V. vinifera* diversity [14]. However, an association panel optimized to capture the largest part of worldwide genetic and phenotypic diversity is still missing for exhaustive exploration of genetic determinism of numerous agronomic traits and genotype by environment interactions.

Our first objective was to design a panel of cultivars suitable for GWAS, starting from 2486 unique cultivars in the grapevine germplasm collection maintained in Institut National de la Recherche Agronomique (INRA) Vassal. We used an original approach to take into account the existence of three genetic pools of cultivars while minimizing relatedness and retaining the main founders of modern cultivated grapevine. Our second objective was to evaluate the diversity captured by this panel using 20 SSR markers and five phenotypic traits. Our third objective was to analyze the effect of various factors on the power achieved by our panel for association tests, by estimating (i) linkage disequilibrium extent using 372 SNPs from four different 2 Mbp genomic regions and (ii) power to detect associations for traits varying in heritability and QTL effects. In addition, we studied diversity and LD in a sample of wild *V. vinifera*, to explore the possibility of performing GWAS in the wild compartment.

## Methods

### Plant material

All plant material was collected at the Vassal repository (French National Grapevine Germplasm Collection, INRA Domaine de Vassal, 34340 Marseillan-Plage, France [50]). This public national collection provides access to any plant material maintained, which is registered as living accessions with accession and cultivar numbers (IDs). All accession information, including ID and passport data, is freely available on the Vassal website. In this study, all tables listing plant material include these IDs.

The experimental research reported here complies with institutional, national, and international guidelines concerning plant genetic repositories. No sample was collected in the wild for this study. All the wild accessions mentioned are ex situ accessions maintained in the Vassal repository. The required Material Transfer Agreement (MTA) was signed by the Director of the Vassal repository, authorising us “to use and store this material for research, experimentation, selection and training purposes”.

#### SNP discovery panel

For SNP discovery, we used sequencing data for a total of 30 accessions (Additional file 1) including: i) a set of 21 cultivars, corresponding to a subset of the G-24 core collection defined by Le Cunff et al. [45], ii) three other cultivars of economic interest (Sultanine, Syrah, Muscat à petits grains blancs) and iii) six accessions of the wild relative *V. vinifera* subsp. *sylvestris*, chosen for their typical wild SSR and morphological profiles. The grapevine genotype PN40024 used for the reference sequence [41] was added as a control.

#### Association panel

**Fig. 1:**
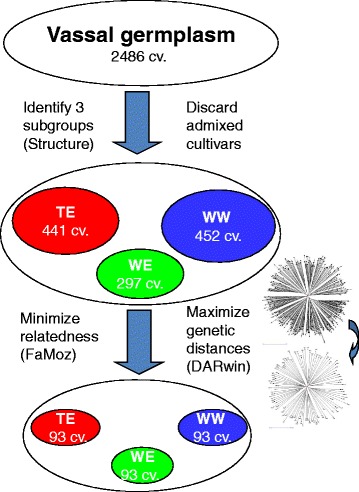
Schematic representation of the method used to design the association panel. WW: wine West, WE: wine East, TE: table East

We sampled an association panel of 279 cultivars selected from 2486 unique cultivars in the Vassal repository, following a procedure taking into account the genetic structure within the collection and minimizing relatedness between cultivars (Fig. 1). First, we assessed the genetic structure within the collection using 20 SSR data from Laucou et al. [51]. We discarded cultivars with more than 20 % missing data and we used the STRUCTURE v2.1 software [52, 53] with the following settings: five independent runs were performed for each *K* value ranging from 1 to 10 by 1, assuming admixture and correlated allele frequencies, with a burn-in phase of 5 × 10^5^ iterations, and a sampling phase of 5 × 10^5^ replicates. We retained the *K* = 3 subdivision, which was relevant according to Evanno’s method [54], as found by Bacilieri et al. [15]. This subdivision matched with the present knowledge about grapevine usage (table *vs* wine) and geographical origin (East *vs* West) [12, 15–17], while resulting in subgroups large enough for further sampling within each subgroup. Second, from the 2276 cultivars left, we selected 1190 non- or low-admixed cultivars, belonging to one of the three subgroups (wine East, WE; wine West, WW; table East, TE) with a membership higher than 80 % according to STRUCTURE results. Third, within each of the three subgroups of this set, we identified the founding individuals as the ancestral or most widely used genitors. This identification was based both on historical and ampelographic knowledge, and on SSR-based relatedness analysis [18], following Lacombe [55]. We then complemented each subgroup up to 93 cultivars, using the Max Length Subtree procedure implemented in DARWin software [56], which allowed well-balanced maximization of the genetic distance between cultivars. For this procedure, we used an Unweighted Neighbor Joining tree based on the DARWin simple matching dissimilarity matrix between the 1190 non- or low-admixed cultivars. We finally removed the remaining first degree related cultivars using FaMoz [57] and ML-Relate [58]. We repeated these last two steps until we obtained a panel with three subgroups of 93 cultivars each.

#### Wild panel

A wild panel was also selected among the accessions of *V. vinifera* subsp. *sylvestris* available in the Vassal collection. After genotyping at 20 SSRs following Laucou et al [51] and careful exclusion of any possibly remaining inter-specific hybrids, 94 accessions (from eight different countries, mainly France), collected in a total of 48 locations, were selected to maximize both the number of geographical origins and the SSR genetic diversity using the Max Length Subtree procedure of DARWin software as described above for cultivars (Additional file 2). Due to loss of weak plants in the greenhouse, only 62 individuals from 34 locations finally composed the wild panel.

### Molecular analyses

#### DNA extraction

DNA was extracted from 200 mg of fresh young leaves or wood collected in the Vassal repository, using the DNeasy Plant Mini or Maxi Kit (Qiagen, Germany) according to the manufacturer’s instructions except that 1 % of polyvinylpyrrolidone (PVP 40,000) and 1 % of β-mercaptoethanol were added to the AP1 buffer. DNA was quantified with Quant-it Picogreen dsDNA Assay Kits (InVitrogen, LifeTechnologies).

#### SNP discovery

SNP discovery was performed in four genomic regions of *ca.* 2 Mb each (Table 1), harboring QTLs for agronomic traits: tannin content and composition on chromosome 8 [24], downy mildew resistance on chromosomes 9 and 12 [59] and berry weight on chromosome 17 [25]. Primer pairs were automatically designed in exons [60] to amplify one specific amplicon of 400–1400 bp per gene, using an automated pipeline combining SPADS v1.0 [61] and PRIMER3 v2.3.6 [62] softwares (detailed procedure available upon request). Within each genomic region, 55–60 amplicons were selected to optimize sequencing (longest possible exon in one direction, absence of microsatellite and poly-T patterns). Small distances between neighbor genes were favored (Additional file 3) to ensure that such distances were sufficiently represented. In addition, to estimate kinship between individuals, 169 amplicons regularly distributed over the whole genome were selected using a similar procedure.

**Table 1:** Number of sequenced amplicons and genotyped SNPs

Region^a^	Number of sequenced amplicons	Number of final amplicons^b^	Mean number of sequenced bp aligned per final amplicon	Total number of SNPs selected for genotyping	Total number of SNPs successfully genotyped
chr8:14529243..16762721	55	41	633	144	86
chr9:3040957..5046544	60	43	629	153	97
chr12:18728014..20687449	60	33	566	147	80
chr17:5195037..7207967	55	48	650	150	109
Distributed over the genome	169	129	657	174	129
Total	399	294	–	768	501

^a^Position in bp on grapevine reference sequence assembly version 12X.0 [69]. Study regions were covered by a single scaffold on chromosomes 8, 9 and 17, by two scaffolds on chromosome 12

^b^Number of amplicons containing successfully genotyped SNPs

For the discovery panel with 30 accessions, a total of 399 amplicons were sequenced in one direction, using the high-throughput Sanger method described by Philippe et al. [63]. Raw sequence files (.ab1) were passed through a pipeline using PHRED and PHRAP [64]. These sequences were then aligned together (not to a reference genome) and SNPs/indels were called, using PREGAP and GAP Shotgun Assembly (with Maximum number of pads = 100 and Maximum percentage of mismatches = 20) within the Staden v4 package [65], followed by manual curation (artifacts, lags)*.* Final validated fasta files (.fas) are publicly available in the SNiPlay database [66, 67] (choose “Grapevine” as species, and “Nicolas_et_al_2016” as project).

#### SNP selection and genotyping

To genotype individuals in the association and wild panels, a total of 768 SNPs were selected, excluding singleton SNPs in the four regions, and distributed SNPs with minor allele frequency (MAF) < 0.2. Priority was given to SNPs with Illumina® scores of 1 (for VeraCode® sequence designability), provided their flanking regions (2x60 bp) produced only single hits using NCBI/BLAST® v2.2.19 [68] against the whole PN40024 reference genome sequence (assembly version 12X.0 [69]). In the four regions, we retained three SNPs per amplicon, over the range of MAF values. For each amplicon distributed over the whole genome, we selected only one SNP with the highest possible MAF value, in order to optimize kinship estimation.

Genotyping was performed using the Illumina® GoldenGate® VeraCode® technology, with two Oligo Pool Assays (OPAs) of 384 SNPs each. After discarding individuals with low genotyping quality, respectively 90, 92, 90 and 62 individuals were retained in WE, WW, TE subgroups and the wild panel (Additional files 2 and 4). Automatic genotype calling was manually checked with Illumina® GenomeStudio v2011.1 software.

### Phenotypic analyses

The phenotypic representativeness of the association panel was assessed for five quantitative traits measured in the Vassal collection (mean values over 2 to 5 years): *véraison* and maturity dates (relative to the reference cv. Chasselas), vigor, berry and cluster weight at physiological maturity. Comparison between the association panel and the whole collection was performed using R packages ‘sm’ v2.2–5.4 [70] for density plots, ‘stats’ v3.0.1 [71] for non-parametric mean equality tests (Wilcoxon rank-sum test), and ‘car’ v2.0–20 [72] for Levene’s variance equality tests. A principal component analysis (PCA) was performed with ‘adegenet’ v1.4–1 R package [73]. We also tested the effect of the association panel subgroup on each quantitative trait by analysis of variance (ANOVA) and Kruskal-Wallis rank sum test using the ‘stats’ R package, with the following model: *Y*_ij_ = *μ* + *S*_i_ + *e*_ij_, where *Y*_ij_ is the phenotypic value of cultivar *j* belonging to subgroup *i*, *μ* the general mean, *S*_i_ the subgroup effect and *e*_ij_ the random effect. Phenotypic data for the association panel are available in Additional file 5.

### Genetic diversity analyses

To assess the genetic representativeness of the association panel, several statistics were computed from the most recent data representing Vassal diversity (genotypes at 20 SSRs for the 2195 cultivars listed in Additional file 4) using GenAlEx v6.501 [74, 75]. For each SSR locus, the number of different alleles (*Na*), effective number of alleles *Ne* = 1/(1-Σ*p*_*i*_^2^) (where *p*_*i*_ is the frequency of allele *i*), observed heterozygosity *Ho* and expected heterozygosity *He* = 1 - Σ*p*_*i*_^2^ were calculated. They were then averaged over the 20 SSRs (data for the association and wild panels are given in Additional file 5). To further assess differences in diversity between subgroups, *Ho*, *He* and MAF were calculated for each SNP locus. All genetic diversity analyses were also performed on the wild panel to allow comparison with the association (cultivated) panel.

### Assessment of population structure and kinship

To check the representativeness of the association panel for genetic structure based on SSR data, a PCA was performed, as implemented in ‘adegenet’ R package. GenAlEx was used to measure pairwise genetic differentiation among subgroups with SSRs or SNPs, using *F*st. Relatedness and the proportion of first degree relationships (parent-offspring + full-sib) were estimated with ML-Relate.

Since genetic structure and kinship may be confounding factors in linkage disequilibrium and genome-wide association studies, corresponding matrices were calculated for the association and wild panels together, i.e. for a total of 334 individuals, based on a combined genotypic file including data for 20 SSRs [51] and 129 SNPs distributed on the genome (this study).

The genetic structure was calculated with STRUCTURE v2.3.1 software. Since STRUCTURE converged very quickly for this sample, we chose a burn-in phase of 5 × 10^4^ iterations and a sampling phase of 5 × 10^4^ replicates, and ran ten replicates of each assumed *K*-level subdivision (from *K* = 2 to 10 by 1). We used the model with uncorrelated allele frequencies and prior geographic information. Both Evanno’s method [54] and the replicates similarity showed that the subdivision in three cultivated subgroups and a wild one was the most probable for the studied sample. The coefficients of membership thus obtained were highly correlated with those obtained for the initial set of 2486 Vassal cultivars with 20 SSRs (Spearman *ρ*^2^ = 0.84: *p*-value < 0.0001). These SSR + SNP coefficients were therefore retained for subsequent corrected LD estimations.

For LD correction by kinship, we used five different co-ancestry estimators, implemented in the CoCoa v1.1 software [76]: i) AIS (Alikeness In State [77]), the probability that the two alleles drawn at a random locus of each of two individuals are identical by state (IBS); ii) WAIS (Weighted Alikeness In State [77]), obtained from AIS by introducing two correction factors to account for the mean probability that two individuals have an IBS allele that is not identical by descent (IBD); iii) BNO [78], which uses a single correction factor for the same goal; iv) LOI [79], a modified correlation coefficient between mean allelic frequencies; v) MLE (Maximum Likelihood Estimator, [80]). For BNO and WAIS, either two or four unrelated groups were assumed, by distinguishing either between the wild and the association panels or between all subgroups (WE, WW, TE, Wild), respectively. When analyzing the four subgroups (WE, WW, TE, Wild) together, the WAIS2 estimator yielded the lowest mean corrected value of inter-chromosomic LD (*r*^2^_VS_ between the SNPs of the four genomic regions, see below) (Additional file 6). Since true LD values between unlinked loci are expected to be null, we selected this estimator for LD correction in all subsequent analyses to minimize bias.

### LD analysis

Linkage disequilibrium was estimated in the four genomic regions between all SNPs with a MAF > 5 %. We used the classical *r*^2^ estimate of correlation between genotypes and two recently developed estimates: one corrected by kinship (*r*^2^_V_) applied to each cultivated subgroup and to the wild panel, and one corrected by both kinship and structure (*r*^2^_VS_) applied to the whole association panel [81]. These corrected estimates were calculated using the ‘LDcorSV’ v1.3.1 R package [81].

The expected LD value within each region was modeled as a non-linear function of physical distance according to Hill and Weir [82] model. LD extent was defined as the physical distance corresponding to an expected LD value of 0.2. The effects of MAF, Nei’s diversity index and annotation features (coding *vs* non-coding, synonymous *vs* non-synonymous) on LD extent were tested with ANOVAs using separate models (detailed in Additional file 7), which included the effects of subgroup and genomic region.

LD landscape within each genomic region was explored: i) through heatmap visualization (‘LDheatmap’ v0.99–1 R package [83]), ii) by plotting mean *r*^2^_V_ against physical position in a 300 Kb-sliding window, with a 10 Kb step, iii) by inspecting the IBS clustering of haplotypes estimated with the localized haplotype cluster model implemented in Beagle v4.0 software [84] using ten iterations.

### Power of the panel for association genetics

We estimated the power of association tests provided by the panel at each SNP according to Rincent et al. [34]. The effects of SNPs on phenotype were tested using the Wald statistic in the framework of the classical mixed model described by Yu et al. [85], which includes a random polygenic effect ***U*** to take into account dependencies between individuals due to relatedness:

$$ Y=\mathbf{1}\mu +{X}_{\mathbf{l}}{\beta}_l+\mathbf{U}+\mathbf{E}, $$

where ***Y*** is the vector of *N* phenotypes, *μ* is the intercept, **1** is a vector of *N* 1, ***X***_**l**_ is the vector of *N* genotypes at the tested locus (0 and 1 corresponding to homozygotes and 0.5 to heterozygotes), *β*_l_ is the additive effect of locus *l* to be estimated, ***U*** ~ *N* (0, ***K****σ*^2^_gl_) is the vector of random polygenic effects with residual polygenic variance *σ*^2^_gl_, ***K*** is the kinship matrix, ***E*** ~ *N* (0, ***I****σ*^2^_e_) is the vector of remaining residual effects with variance *σ*^2^_e_, ***I*** is an identity matrix of size *N*, ***U*** and ***E*** are independent.

We estimated the power to detect association in our panel, at each SNP locus in the four genomic regions. The trait had a known heritability *h*^2^ (0.3, 0.5, 0.7 or 0.9). Each locus had a known effect *β*_l_ explaining a fraction (0.05, 0.1 or 0.25) of additive genetic variance. Kinship *K* between individuals was estimated from molecular markers using different methods described above (AIS, WAIS2, WAIS4, LOI, MLE). To take into account multiple testing at 372 loci, we used a family wise error rate (FWER) value of 0.05. To obtain the corresponding *p*-value threshold, we divided this FWER by the number of independent tests (Meff), estimated according to Li and Ji [86].

## Results

### Diversity and structure of the association and wild panels, assessed with SSRs

**Fig. 2:**
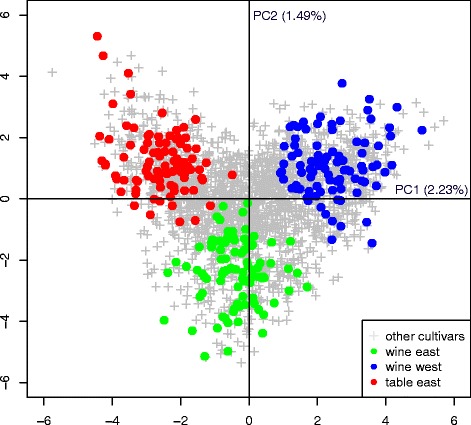
PCA analysis based on 20 SSRs for comparing the association panel with the whole Vassal collection. Other cultivars: the Vassal collection but the association panel

The association panel designed from the Vassal collection, composed of three subgroups of 93 cultivars each (wine East, WE; wine West, WW; table East, TE; Additional file 4), fulfilled the joint objectives of representativeness and low relatedness. The SSR diversity captured in the association panel was representative of the diversity existing in the whole Vassal collection (Additional file 8). The total number of alleles was lower in the panel than in the Vassal collection (246 *vs* 307), with only rare alleles (MAF < 0.05 within the Vassal collection) not retained. SSR allelic frequencies were highly correlated between the panel and the Vassal collection (Pearson *R*^2^ = 0.99). The three panel subgroups accurately represented the three main divisions of the Vassal collection along the first two PCA axes (Fig. 2). Mean relatedness was already low in the Vassal collection (0.047), and it was further reduced in the association panel (0.042; Wilcoxon rank-sum test, *p*-value < 0.0001, Additional file 9). The proportion of first degree relationships was reduced from 0.52 % in the Vassal collection to 0.24 % in the panel.

The wild panel was found less diverse than the cultivated association panel and closest genetically to the wine West subgroup (Additional files 8 and 10).

### Phenotypic diversity captured by the association panel

**Fig. 3:**
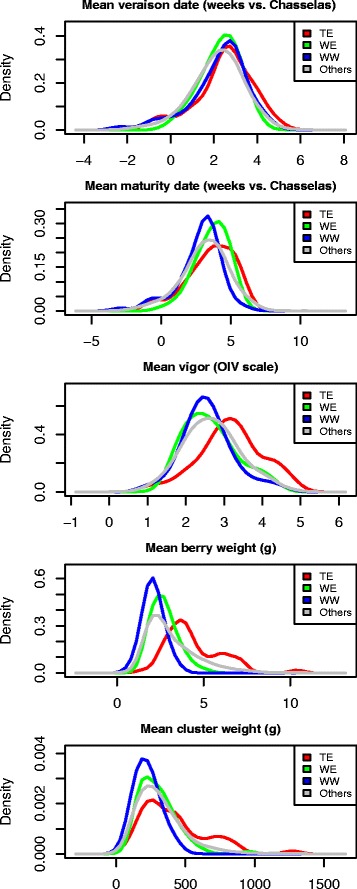
Distribution of five phenotypic traits in Vassal collection and the association panel. WW: wine West, WE: wine East, TE: table East. Others: the Vassal collection but the association panel

The phenotypic diversity within the association panel was representative of the diversity in the whole Vassal collection for the five quantitative traits. The mean trait values in the association panel did not significantly differ from those in the Vassal collection, except for *véraison* date (Fig. 3, Additional file 11). Variance was significantly smaller in the association panel for two traits only (maturity date and berry weight, Additional file 11), for which a very large proportion of variance (between 84 and 96 %) was captured. Moreover, the phenotypic diversity in the panel spanned the whole range of phenotypic variability of the Vassal collection, as illustrated by the PCA plot (Additional file 12).

The panel was structured differently for these traits, according to fruit usage, geography or both. ANOVA and Kruskal-Wallis showed a significant effect of subgroup on phenotypic variation of all traits except *véraison* date (*p*-value < 0.001). Subgroup explained 7, 11, 44 and 18 % of total phenotypic variation (*R*^2^) for maturity date, vigor, berry weight and cluster weight, respectively. For these traits, we also observed significant pairwise differences between subgroup mean values (Fig. 3, Additional file 11).

### SNP discovery and genotyping with OPAs

Out of the 399 sequenced amplicons, 74 % harbored SNPs which could be successfully genotyped on all individuals (Table 1, Additional file 3). In this final set of amplicons, 4584 SNPs were detected for a total of 187,624 bp, i.e. an average of 2.4 SNP per 100 bp. This large diversity is consistent with the previously published values in grapevine [44, 45]. Out of the 768 SNPs selected for panel genotyping, 267 were discarded during manual curation of raw SNP genotype data. Finally, a total of 334 plants were successfully genotyped using 501 SNPs: 372 in the four genomic regions and 129 distributed over the whole genome (Additional file 13).

Selection of SNPs based on sequencing results in the discovery panel proved to be relevant, since MAF values of the 372 SNPs successfully genotyped in the four regions were highly correlated between the discovery and association panels (Spearman *ρ*^2^ = 0.6: *p*-value < 0.0001).

Less than 20 % of the biallelic SNPs found by sequencing the discovery panel met all the selection criteria for genotyping with Illumina® VeraCode®. This deficit arose mainly from polymorphism in SNP flanking sequences, which prevented the definition of Illumina® primers. SNPs were also discarded because of duplication of SNP flanking sequences or too low allele frequency. The selection of 372 SNPs among the 1280 non-singleton SNPs found by sequencing in the four genomic regions, introduced a small bias towards larger MAFs (goodness-of-fit *χ*^2^ test for comparison of both distributions, *p*-value = 0.045, with 97 out of 372 SNPs having a MAF < 0.1 *vs* 491 out of 1280). It also introduced a bias towards exonic regions, with 76 % of the 372 selected SNPs in exons *vs* 31 % of the 1681 initially available SNPs. This unavoidable bias probably resulted from the larger polymorphism found in introns compared to exons, which decreased the occurrence of SNPs with monomorphic flanking sequences required for this genotyping method.

Moreover, despite careful selection of SNPs for genotyping, only 65 % of the selected SNPs yielded high quality genotype data. This additional SNP loss was due to more than three clusters suggesting potential copy number variation (for *ca.* 10 % of discarded SNPs), insufficient cluster separation, small additional cluster, no amplification or monomorphism.

### Diversity of the association and wild panels, assessed with SNPs

The distributions of MAFs and Nei’s diversity indices showed differences among subgroups and genomic regions. For MAFs, differences were significant (Fisher’s exact test) in the three subgroups (*p*-values < 0.02) and for chr08 and chr12 (*p*-values < 0.004). For Nei’s diversity, differences were significant (Fisher’s exact test) in wine East and wine West subgroups (*p*-values < 0.001) and for chr08 and chr17 (*p*-values < 0.002).

Pairwise differentiation between subgroups varied among genomic regions (0.01 < *F*_st_ < 0.09; Additional file 14).

SNP diversity averaged over the four genomic regions was significantly lower in the wild panel than in the association (cultivated) panel, with Nei’s diversity index values of 0.22 and 0.28, respectively (Wilcoxon rank sum test, *p*-value < 0.0001).

### Linkage disequilibrium assessment

#### Comparison of LD extent between subgroups and genomic regions

**Fig. 4:**
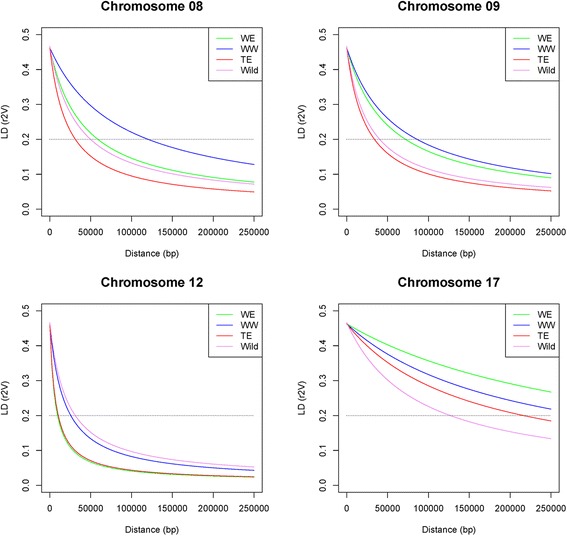
Genotypic LD (*r* ^2^_V_) modeled as a function of physical distance according to Hill and Weir [82]. LD was modeled separately in each subgroup of the association panel and in the wild panel, for each of the four genomic regions

LD extent for a predicted *r*^*2*^_V_ of 0.2 varied from 9 to 458 Kb according to subgroup and genomic region (Fig. 4, Table 2). LD extent over the four genomic regions (*r*^*2*^_VS_) for the whole association panel was 43 Kb. According to this estimate from four genomic regions, the number of markers required to reach an expected *r*^*2*^_VS_ value of 0.45 between any causal polymorphism in the genome and the nearest marker was 476,604, corresponding to one SNP per Kb on average. LD extent differed significantly among genomic regions (ANOVA, *p*-value < 0.01), but not among subgroups (Additional file 7). MAF and Nei’s diversity index significantly affected LD extent (ANOVA, *p*-value < 0.01), whereas annotation features (coding *vs* non-coding, synonymous *vs* non-synonymous) did not (Additional file 7).

**Table 2:** LD extent (*r* ^2^_V_) in each of four subgroups and four genomic regions. Expected LD threshold was 0.2. WE (wine East), WW (wine West) and TE (table East) are the three subgroups of the association panel

Study region	Linkage disequilibrium extent				Genetic size of the region (cM)^a^
	WW	WE	TE	wild panel	
chr 8	120 Kb (72)	59 Kb (79)	31 Kb (56)	49 Kb (50)	11
chr 9	86 Kb (75)	71 Kb (73)	33 Kb (78)	40 Kb (57)	15
chr 12	25 Kb (59)	9 Kb (63)	10 Kb (62)	31 Kb (46)	3
chr 17	295 Kb (80)	458 Kb (67)	210 Kb (79)	127 Kb (77)	12

^a^Estimated from the composite map of Doligez et al. [40]

The number of SNPs with MAF ≥ 5 % is given in parentheses

#### Comparison of LD landscape between subgroups and genomic regions

**Fig. 5:**
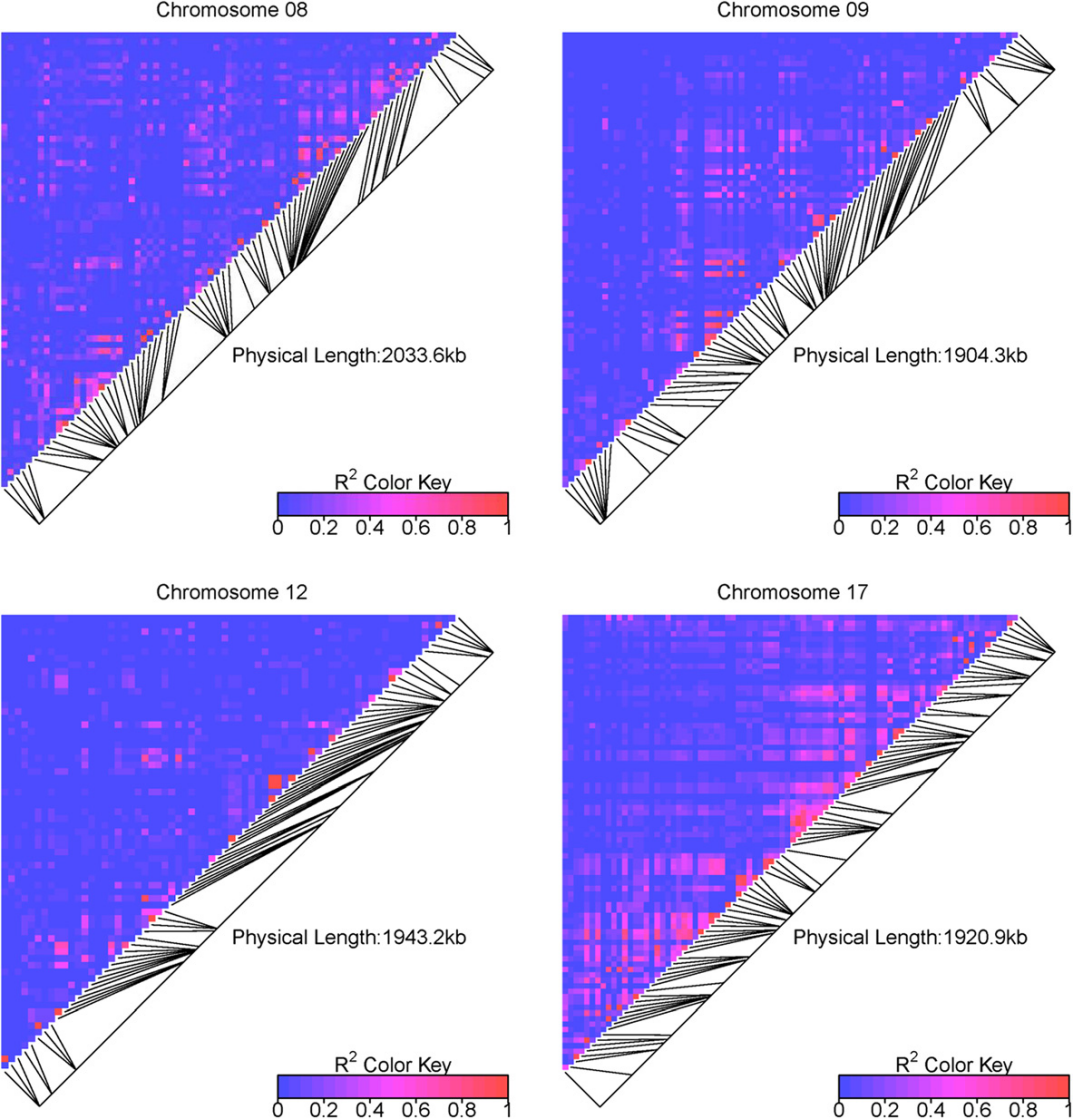
Heatmaps of genotypic LD (*r* ^2^_VS_) in four genomic regions in the whole association panel

The heatmaps of all pairwise *r*^2^_VS_ values showed that the detailed LD pattern along each genomic region in the association panel was highly variable (Fig. 5). Mid-level *r*^2^_VS_ values (~0.5) were found between SNPs as far as 500 Kb apart in some regions (e.g. on chr09 and chr17) whereas there was no LD between adjacent blocks of SNPs in other regions (e.g. on chr17 again).

**Fig. 6:**
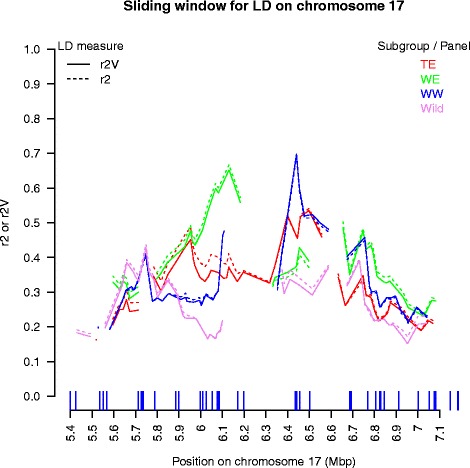
Mean local LD (*r* ^2^_V_) in a 300 Kb-sliding window along the chromosome 17 genomic region. Local LD was computed separately in each subgroup of the association panel and in the wild panel. Only mean LD values based on at least ten marker pairs are plotted. Vertical lines on the *x*-axis indicate SNP positions

Sliding window analysis revealed a mean local LD very different among genomic regions (from *ca.* 0.1 to 0.7), with a different ordering of subgroups (Fig. 6, Additional file 15). Some genomic regions consistently showed low or elevated LD levels in all subgroups (e.g. on chr08 and chr17, around 15.5 and 6.4 Mbp, respectively), while others harbored large differences in local LD among subgroups (e.g. on chr17 around 6.0–6.1 Mbp). Part of mean local LD was explained by mean local inter-SNP distance (*R*^2^ of linear regression of mean LD on mean inter-SNP distance in each window explored = 0 to 52 %, depending on genomic region), but the part explained was > 20 % in only five of the 16 subgroup x chromosome combinations. Local LD showed no particular relationship with local diversity (Nei’s index) (Additional file 16). Interestingly, larger local differentiation between cultivated subgroups and the wild panel was observed on chr17, especially around 5.7 Mbp (Additional file 16), co-localized with large differences in local LD between subgroups.

Haplotypic structures were very different between genomic regions (Additional file 17), with especially large haplotypic blocks on chr09 and chr17.

### Power of panel for association studies

We assessed the power of association tests provided by the panel at 372 SNPs within the four genomic regions, with different trait heritabilities, a variable part of additive genetic variance explained by SNPs, five different kinship estimators and a family wise error rate (FWER) of 5 % divided by the estimated number of independent loci (Meff = 217).

Whatever trait heritability and locus effect, AIS kinship estimator resulted in the highest power to detect association, with a difference in mean power reaching 25 % between AIS and WAIS4 for high heritability and large locus effect (Additional file 18).

**Fig. 7:**
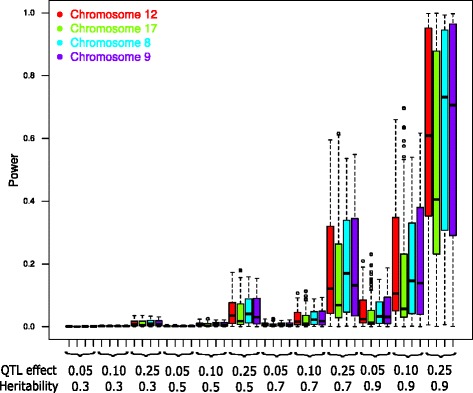
Variation of power distribution within four genomic regions (at a total of 372 SNPs). We used a family wise error rate of 5 %, AIS kinship estimation and various heritability and QTL effect values

Power variation between loci was mainly explained by heritability, QTL effect, and allele frequency. As expected, power increased with heritability, for a given part of genetic variance explained by the locus, whatever the kinship estimator (Additional file 18) or genomic region (Fig. 7). For a locus explaining 25 % of genetic variation, mean power over the 372 SNPs with AIS estimator varied from 1 to 59 % when heritability varied from 0.3 to 0.9. Power also increased with QTL effect, for a given heritability value. Relaxing FWER from 5 to 10 % led to increased mean power (e.g. with AIS, for *h*^2^ = 0.7, at a locus explaining 25 % of genetic variation, power was 22 % with FDER = 0.1 *vs* 18 % with FDER = 0.05).

We observed a large variation of power among loci, which markedly increased with both heritability and genetic variance explained by the locus (Fig. 7 and Additional file 19). As expected, power greatly increased with MAF. Detection power for loci with MAF > 25 % and strong effect (0.25) could reach 95 % for a highly heritable trait with AIS (Additional file 19).

Power was quite similar between the different genomic regions, except for chr17, which showed the lowest power whatever the kinship estimation method. Except for AIS kinship, this difference was no longer observed when removing loci with MAF < 5 % (data not shown), indicating that it mostly originated from the higher proportion of rare alleles found in the chr17 region. It could also result from lower local differentiation among the three panel subgroups on chr17 (Additional file 14).

Power at a marker linked to a causal locus logically decreased according to LD between the marker and this locus; it could reach 31 % for an expected *r*^2^_VS_ value of 0.45, with *h*^2^ = 0.9 and a QTL effect of 25 %, when using AIS estimation and MAF > 5 % (Table 3).

**Table 3:** Power of association tests at markers linked to causal polymorphims according to LD extent in the association panel, heritability and effect of causal polymorphism (% of trait variance explained)

*r* ^2^_VS_^a^	Mean power at the marker linked to causal polymorphism^b^					
	*h* ^2^ = 0.5		*h* ^2^ = 0.7		*h* ^2^ = 0.9	
	effect = 10 %	effect = 25 %	effect = 10 %	effect = 25 %	effect = 10 %	effect = 25 %
1	0.9	6.0	3.4	22.4	22.9	69.6
0.1	0.1	0.6	0.3	2.2	2.3	7.0
0.15	0.1	0.9	0.5	3.4	3.4	10.4
0.2	0.2	1.2	0.7	4.5	4.6	13.9
0.25	0.2	1.5	0.9	5.6	5.7	17.4
0.3	0.3	1.8	1.0	6.7	6.9	20.9
0.35	0.3	2.1	1.2	7.8	8.0	24.4
0.4	0.4	2.4	1.4	8.9	9.2	27.8
0.45	0.4	2.7	1.6	10.1	10.3	31.3

^a^Squared correlation between the causal polymorphism and the linked marker, corrected by kinship and structure

^b^Mean power over the 314 SNPs with MAF > 5 % in the four genomic regions, assuming a family wise error rate of 5 % and using AIS kinship estimation, calculated as the power at the causal locus multiplied by the corrected LD between the causal locus and the marker

## Discussion

### Design of the association panel

#### Global genetic and phenotypic diversity captured in the association panel

Our panel captured a large part of the genetic and phenotypic diversity present in the Vassal collection. The panel contains all the non-rare alleles (MAF > 0.05) at 20 SSR loci and shows a similar level of genetic diversity, as compared with Vassal collection (*He* = 0.78 in the panel *vs* 0.77 in Vassal). We also observed the same distribution of non-rare allele frequencies and similar phenotypic means and variances. The Vassal collection, which includes 2344 unique *V. vinifera* cultivars [18], is to date the largest and the most diverse and comprehensive collection of cultivated grape worldwide. The other largest *V. vinifera* collections in the world are those of Encin (IMIDRA, Spain) with 1852 cultivars [87], Conegliano (CREA-VIT, Italy) with 1320 cultivars (CREA-VIT, personal communication), Geilweilerhof (JKI, Germany) with 1136 cultivars (E. Maul, personal communication), FEM (Fondazione Edmund Mach, Italy) with 733 cultivars [16] and USDA (USA) with 583 cultivars [14]. Moreover, the Vassal collection had already been curated for homonymies and misnamings, phenotyped for several years [88] and entirely genotyped with 20 SSRs [51]. It was therefore a starting material of choice to derive a widely useful association panel.

#### Advantages of the method used to design the association panel

An original method was used to design our grapevine association panel. Our approach took into account the long-term historical genetic structure shaped by human selection for contrasting uses (table *vs* wine) and geographic adaptation (East *vs* West). In these three genetic pools, we selected key founder cultivars of modern cultivated germplasm and removed closely related genotypes (first-degree relatives). This method yielded a sample with characteristics more appropriate for association genetics than core collections previously defined from the Vassal collection, in addition to its larger size (279 *vs* 141 and 92 for the morphological and genetic core collections, respectively) [38, 45].

This method ensured a balanced representation of all three major genetic pools in the final panel, by taking into account the genetic structure of the whole collection. In contrast, the previously defined genetic core collection [45] over-represented the table East (TE) genetic pool (Additional file 10). This resulted from the combination of the larger diversity present in the TE genetic pool and the sampling method used, which maximizes the number of alleles [89, 90]. Since the three major genetic pools correspond to different uses (table *vs* wine) and agro-climatic conditions (Eastern *vs* Western Europe), different alleles of interest have probably been selected among pools. Therefore, their balanced representation in the panel is crucial to ensure sufficient power of association tests for potentially involved alleles.

Our method also succeeded in limiting relatedness in the final sample, by decreasing it within each subgroup. This is essential in grapevine, where the large majority of cultivars in collections (75–80 %) are closely related by a first-degree relationship [14, 18]. Our panel therefore combined limited relatedness with the low structure derived from the three *V. vinifera* genetic pools. For association mapping, the ideal sample according to Yu et al. [85] should have minimal structure and relatedness, in order to yield the largest QTL detection power for traits not correlated with structure. For species where such samples are difficult to obtain, two main alternative types of designs are possible: i) samples with structure and/or relatedness, which require controlling for false positives in association tests, or ii) family-based designs with controlled structure. For instance, the latter possibility was applied to apple, with a large factorial design involving six parents [91]. In that case however, diversity and recombination number remained limited. More recently, NAM and MAGIC designs, based on larger parental diversity and more recombination cycles, have been developed in maize, barley and wheat [92–95]. They represent an interesting compromise between association and linkage approaches, enabling the study of traits correlated with genetic structure in germplasm panels, even though to our knowledge power has not been compared between NAM or MAGIC and germplasm panels. In grapevine, creating such material would take a very long time, even using short cycle material (microvine [96]), and would be very costly due to the space needed to maintain the numerous plants required. Therefore, the association panel we selected from the largest germplasm collection already available is meanwhile probably the best solution, given its diversity, structure and relatedness features. Controlled crosses could be used in complement to study the genetic determinism of traits correlated with genetic structure, with intermediary heritability, or with low frequency and/or effect of functional alleles [29].

In addition, our method allowed recovering the same level of genetic diversity as in the Vassal and genetic core collections (*He* around 0.8), without the need to retain rare alleles as in core collections. This offers an advantage for association genetics, where the use of rare alleles is not recommended due to poor power and variance estimation [97]. Moreover, our method yielded a non-rare allele frequency distribution in the panel not significantly different from the Vassal and genetic core collections (Fisher's exact test, *p*-value = 0.9718 for panel *vs* Vassal, *p*-value = 0.6184 for panel *vs* core collection).

#### Comparison with association panels of other species

Our sampling method is thoroughly described and thus applicable to other species. By contrast, sampling methods are rarely described in published reports of other plant diversity association panels. Individual plants composing the sample are empirically chosen to try to best represent the diversity available in germplasm collections and/or breeding programs, often based on pedigree information, but without relying on an objective quantitative method. Only a few studies mention the definition of core collections (e.g. [98] in apple, [99] in sunflower) or other methods such as pedigree analysis followed by the calculation of individual weighted contributions in soybean [100].

Our association panel of 279 cultivars selected from a germplasm collection is the largest ever defined in a perennial fruit crop. In fruit trees, since breeding programs are lengthy, there are usually far less unique genotypes available in germplasm collections than for annual crops (e.g. hardly more than 2000 in grape *vs* 50,000 in rice) and a large part of these genotypes are closely related [101]. It is therefore crucial to optimize the design of association panels in such crops, as it takes a very long time to reach fruit set in a field trial. Moreover, multi-site trials are probably the best alternatives to the problematic application of controlled abiotic stresses in field trials with unwieldy plants. These difficulties may explain both the very low number of association studies in fruit trees to date, compared to forest trees and annual crops, and the small size of association panels already defined in fruit trees (always less than 200, more often around 100).

The size of our association panel is comparable to the size of intra-specific diversity association panels in forest trees (considering unrelated accessions only) or annual crops, despite the above mentioned drawbacks specifically linked to grape perennial status. In forest trees, a few panel sizes were above 500, as for Douglas fir [102] and loblolly pine [103], but most were between 100 and 450. In annual crops, panels most frequently contained between 150 and 400 accessions, with the largest reaching about 500 accessions in maize [104], rice [105] and spring wheat [106]. Since increasing sample size is one of the possible ways to increase detection power [107], more variants with small effect or frequency could be detected with a larger panel. However, we would probably not be able to further increase our panel size without concurrently increasing relatedness, unless new genetic resources were included (notably from Eastern Europe [15]).

Our association panel has a very low genetic structure (pairwise *F*_st_ between subgroups < 0.09), which is an advantage since structure is a confounding factor in association genetics. The low genetic structure already present in the Vassal collection (Fig. 2) was maintained while designing the panel, despite our discarding individuals admixed between genetic pools. By contrast, panels of other crops are sometimes much more structured, with up to a dozen subgroups as in sorghum or maize [33, 108] and some pairwise *F*_st_ estimates as large as 0.4, in rice for example [109]. In our panel, the part of phenotypic variance explained by genetic structure varied among traits from a few percent to more than 40 %, in the same range as in rice or maize [104, 109].

#### Limitations of the association panel

The first limitation of our association panel is that it is exclusively composed of *V. vinifera* individuals. It will indeed be a valuable tool to search for alleles of interest for quality, phenology and yield-related traits. Nevertheless, other species in the *Vitis* genus exhibit larger variation for disease resistance or adaptation to environmental stresses. Therefore for these traits, association panels will also have to be specifically designed for other *Vitis* species.

A second limitation arises from the relationship between genetic and phenotypic structure for some traits, due to differential fixation of alleles among subgroups following diversifying selection and/or genetic drift. In such cases, some marker-phenotype associations will not be detectable in association tests based on mixed models correcting for structure. Differentiation among subgroups will thus need to be considered [110] and associations tested within subgroups. A phenotypic wine-table and/or East-West structure was observed in the panel for most traits. However, the percentage of total phenotypic variation explained by genetic structure was low (less than 20 %), except for berry weight (44 %). Moreover, as discussed above, genetic structure is limited in our panel.

### LD extent

This study showed that LD between SNPs in grapevine may extend further than previously reported. In the association panel, decay of expected LD down to 0.2 varied among subgroups and genomic regions, from 9 to 458 Kb (Table 2). This was larger than both the value of *ca.* 250 bp reported by Lijavetzky et al. [44] and the value of less than 10 Kb given by Myles et al. [14]. Several hypotheses may explain this discrepancy. First, LD extended further on chr17 study region than in the other regions. Second, previous reports were genome-wide studies while ours is based on four regions only. Third, LD extent was estimated in different ways: Hill and Weir’s model in our study, non-linear regression in Lijavetzky et al [44], bin medians in Myles et al [14]. Fourth, in Lijavetzky et al [44], only intra-genic LD was measured, which might have influenced the regression curve. Last, the possibility of some bias in our study due to the small number of SNPs in some genomic region x subgroup combinations cannot be ruled out (Table 2).

Variability in LD extent essentially resulted from differences between genomic regions. Differences between subgroups were much more limited, even though LD extent was lowest in the table East subgroup, which is consistent with the larger number of generations that have occurred in this genetic pool [18]. The most noticeable feature was the larger LD extent on chr17 compared to other genomic regions. The excess of SNPs with small MAF in this region was not sufficient to explain this discrepancy, since ANOVA showed independent effects of MAF and genomic region on LD extent (Additional file 7). In the association panel, the larger LD extent in this region of chr17 is more probably due to selection for berry size during and after domestication. Indeed, the larger cultivated-wild differentiation also observed in this region coincided with the candidate domestication locus reported by Myles et al. [14] and the berry weight QTL reported by Doligez et al. [25]. We showed in this latter study that the region harbored a grapevine gene from a family probably involved in fruit size changes during tomato domestication. However, this assumption cannot explain the larger LD extent also observed in the wild panel in this region.

In the wild panel, LD extent ranged from 31 to 127 Kb and was not significantly different from the association (cultivated) panel, suggesting equivalent possibilities for GWAS. Moreover, with both SSR and SNP markers, we found a lower diversity in the wild sample than in the cultivated one, which is not expected in crops. Our data therefore seem to reinforce the hypothesis that no strong bottleneck occurred during grapevine domestication events, as argued by Myles et al. [14]. Our results could also illustrate the genetic erosion undergone by the wild compartment, probably mainly due to anthropic pressure on natural habitat and biotic stresses, in particular phylloxera and mildews since the middle of the nineteenth century. In addition, the wild sample contains only Western accessions, which could also result in lower diversity.

Our LD analysis offers several advantages over previous ones in grapevine. First, the sample size is larger than in Barnaud et al. [38, 39] and Lijavetzky et al. [44], with subgroups of sufficient size to prevent any LD overestimation due to small sample size [111]. Second, we estimated LD separately in each of the three main diversity groups, using adequate samples, and could therefore compare LD extent between subgroups, which had never been reported before. No significant difference was found, which is consistent with the relatively short history of cultivated grapevine in terms of recombination [13, 14, 112]. Third, we used novel LD estimates allowing correction for structure and/or kinship [81]. Kinship correction appeared useful in our case, whereas structure correction did not. This was probably partly due to the very low overall genetic differentiation in cultivated *V. vinifera*, but also to the fact that the structure between wild and cultivated samples was already taken into account through the kinship estimation method used (WAIS assuming two unrelated groups). Last, the genotyping methodology used, coupled with manual curation of raw data yielded highly reliable genotypes. This is clearly an advantage over fully automated high throughput methods such as the one used in Myles et al. [14].

Although it is particularly difficult and debatable to compare LD extent among species, our results indicate that LD decays far less rapidly in grapevine than in forest trees [113, 114], less rapidly than in *Arabidopsis* [115], and at a comparable rate to that in maize [116] or rice [105].

LD extent variability could be partly explained by MAF and Nei’s diversity but not by annotation properties. A large part of LD variation is still linked to unknown causes of variation among genomic regions, probably related to local selection or to differences in recombination rate or genomic structure.

### Power achieved by the association panel

Since we estimated power at each locus considering that this locus was a causal mutation, we assessed the maximal power of our panel. When all individuals are not genotyped for all polymorphisms, panel power to detect associations depends on linkage disequilibrium between genotyped SNPs and causal mutations. By combining maximal power and expected extent of LD corrected for stratification, we obtained an estimation of power for markers linked to causal polymorphisms for different trait heritabilities and QTL effects (Table 3). Given the variability in the size of haplotype blocks along the genome (Additional file 17) and in MAF (Additional file 18), local power may vary significantly around these mean values.

We observed large variation in mean power depending on the kinship matrix used in the mixed model, with a difference in power reaching 25 % between AIS and WAIS4 kinship estimators. AIS always yielded the highest power. This may result from a lower correlation of global kinship (estimated from markers spread across the genome) with local kinship (estimated from a single marker), which leads to increased power as shown by Rincent et al. [34].

The method we used for multiple testing correction (dividing family wise error rate by the number of independent loci) is quite conservative, although much less than the Bonferroni method. Using false discovery rate could be an interesting alternative to take into account multiple testing.

## Conclusions

We defined and characterized an association panel offering the best operational representation of diversity so far for association genetics in cultivated *V. vinifera*. Our estimates of LD and power of association tests in four genomic regions suggest that at least half a million SNPs will be required for efficient GWAS in this panel. Forthcoming genome-wide genotyping based on the 18 K Illumina® chip [117] or GBS (genotyping by sequencing) on this panel will soon allow a more exhaustive estimation of the range of marker density needed.

This panel achieves reasonable power to detect associations between traits with high heritability (> 0.7) and loci with intermediate allelic frequency (> 10 %) explaining a large part of genetic variance (> 10 %). This study illustrates that simulating power of an association panel on all or a subset of polymorphisms before conducting GWAS, as in Rincent et al. [33], is very useful to rationally choose: (i) traits that can be evaluated in trials, knowing their heritability; (ii) the MAF threshold for removing markers that increase stringency through multiple testing correction without improving panel power; (iii) the best kinship estimator to detect associations without increasing false positive rate; (iv) the type and stringency of correction method for multiple testing.

Highly precise phenotypic data are required for powerful association genetics. The association panel thoroughly characterized in this study is a valuable resource to be established in multi-site experimental trials. One research group is presently phenotyping this panel in a greenhouse in North-Eastern France and another group has planned to set up this panel in South America. This panel could also be useful for genomic selection evaluation, due to its maximized diversity [118], and it could even serve as a « universal » training population in *V. vinifera* genomic selection [119].

## Availability of supporting data

All plant material is available from the Vassal public repository, and accession information, including ID and passport data, is available on its website [http://www1.montpellier.inra.fr/vassal]. Most datasets supporting the conclusions of this article are included within the article and its additional files, in the SNiPlay repository [http://sniplay.southgreen.fr/cgi-bin/SNPqueries_v3.cgi], in Lacombe et al. [18], in the references cited therein, or in the European *Vitis* database [http://www.eu-vitis.de/index.php]. The SNiPlay repository harbors sequence and variation data for several southern and Mediterranean plant species. Although the remaining SSR and phenotypic data will not be widely released before complete analysis, these data can be provided upon request.

## Additional files

Additional file 1: Table S1. Resequencing sample of 30 *Vitis vinifera* individuals for SNP discovery. (XLSX 10 kb) Additional file 2: Table S2. List of the 62 accessions of *Vitis vinifera* subsp. *silvestris* composing the wild panel. (XLSX 13 kb) Additional file 3: Figure S1. Position of the amplicons selected in the four LD study regions for SNP discovery by sequencing. For amplicons in red, at least one SNP was successfully genotyped with Illumina® VeraCode®. (PPTX 89 kb) Additional file 4: Table S3. List of the 2195 cultivars representing the Vassal collection used for computing diversity indices. (XLSX 102 kb) Additional file 5: Table S4. Phenotypic data for five traits and genotypic data at 20 SSRs, for the association panel. Genotypic data at 20 SSRs for the wild panel. (XLSX 69 kb) Additional file 6: Figure S2. Comparison of the distribution of the *r* ^2^_VS_ LD values between unlinked SNPs of the four genomic regions, corrected by both structure and one of seven kinship matrices, for the whole association panel. The boxplot for *r* ^2^ is the uncorrected reference. (PDF 76 kb) Additional file 7: Table S5. Effect of subgroup, genomic region, MAF, diversity, and SNP annotation features on LD (*r* ^2^_V_) extent. The three subgroups of the association panel and the wild panel were included. Pairs of SNPs were classified as having: 0, 1 or 2 SNPs with MAF < 0.2; 0, 1 or 2 SNPs with Nei < 0.3; 1 or 2 SNPs in exons. Pairs of SNPs in exons were classified as having 0, 1 or 2 synonymous SNPs. (XLSX 11 kb) Additional file 8: Table S6. SSR diversity indices in the Vassal collection, the association panel and the wild panel, averaged over 20 SSRs. *Na*: number of alleles, *Ne*: effective number of alleles, *Ho*: observed heterozygosity, *He*: expected heterozygosity. The values are means ± standard error. The superscript letters indicate the result of the Wilcoxon rank-sum test for bilateral comparisons of means. (XLSX 9 kb) Additional file 9: Figure S3. Histogram of relatedness in the Vassal collection *vs* in the association panel, based on 20 SSRs. (PDF 4 kb) Additional file 10: Figure S4. PCA analysis of the Vassal collection and wild panel, based on SSR data. Relative position of: A. The three subgroups of the association panel. B. The genetic core collection. (PDF 254 kb) Additional file 11: Table S7. Comparison of phenotypic mean and variance for five traits between the association panel subgroups. (XLSX 490 kb) Additional file 12: Figure S5. PCA analysis for comparing the association panel with the whole Vassal collection, based on five quantitative traits. (PDF 86 kb) Additional file 13: Table S8. List of the 501 SNPs successfully genotyped. (XLSX 103 kb) Additional file 14: Figure S6. Pairwise genetic differentiation (*F*_st_) amongst subgroups (based on SNPs or SSRs). All *F*_st_ values were significantly different from zero according to the test implemented in GenAlEx, based on 1000 permutations. WW: wine West, WE: wine East, TE: table East. (PDF 8 kb) Additional file 15: Figure S7. Mean local genotypic LD in a 300 Kb-sliding window along the genomic regions on chromosomes 8, 9 and 12 in each subgroup of the association panel (WE, WW and TE) and the wild panel. Only mean LD values based on at least ten marker pairs are plotted. Vertical lines on the *x*-axis indicate SNP positions. (PDF 19 kb) Additional file 16: Figure S8. Mean local Nei’s diversity (A) and *F*_st_ (B) in a 300 Kb-sliding window along the genomic regions on chromosomes 8, 9, 12 and 17 for each subgroup of the association panel (WE, WW and TE) and the wild panel. Only mean values based on at least five markers are plotted. Vertical lines on the *x*-axis indicate SNP positions. (PDF 39 kb) Additional file 17: Figure S9. IBS clustering of reconstructed haplotypes in the four genomic regions. (PDF 4111 kb) Additional file 18: Figure S10. Variation of power distribution at 372 SNPs using five different kinship estimators. We used a 5 % family wise error rate and various heritability and QTL effect values. (PDF 19 kb) Additional file 19: Figure S11. Variation of power according to minor allele frequency (MAF), with a 5 % family wise error rate and using AIS kinship, for different levels of heritability and QTL effect. (PDF 236 kb)

## Abbreviations

AIS: alikeness in state
ANOVA: analysis of variance
ANR: Agence Nationale de la Recherche
BNO: Bernardo
bp: base pair
chr: chromosome
CNG: Centre National de Génotypage
CNIV: Comité National Interprofessionnel des Vins d’appellation d’origine
CREA-VIT: Centro di Recerca per la Viticoltura
cv.: cultivar
DNA: deoxyribonucleic acid
EPGV: Etude du Polymorphisme des Génomes Végétaux
FEM: Fondazione Edmund Mach
FWER: family wise error rate
GBS: genotyping by sequencing
GWAS: genome-wide association study
IBD: identical by descent
IBS: identical by state
ID: identification number
IMIDRA: Instituto Madrileño de Investigacion y Desaroll Rural, Agrario y Alimentario
INPT: Institut National Polytechnique de Toulouse
INRA: Institut National de la Recherche Agronomique
JKI: Julius Kühn Institut
Kb: kilo base pair
LD: linkage disequilibrium
LOI: Loiselle
MAF: minor allele frequency
MLE: maximum likelihood estimator
MTA: Material Transfer Agreement
OPA: oligo pool assay
PCA: principal component analysis
QTL: quantitative trait locus
SNP: single nucleotide polymorphism
SSR: simple sequence repeat
TE: table east
USDA: United States Department of Agriculture
WAIS: weighted alikeness in state estimator
WE: wine east
WW: wine west
