# Supplementary figures and images for: Genetic diversity, linkage disequilibrium and power of a large grapevine (Vitis vinifera L) diversity panel newly designed for association studies
Source: BMC Plant Biol. 2016 Mar 22;16:74. doi: 10.1186/s12870-016-0754-z (PMC4802926; doi:10.1186/s12870-016-0754-z)

## Slide 1
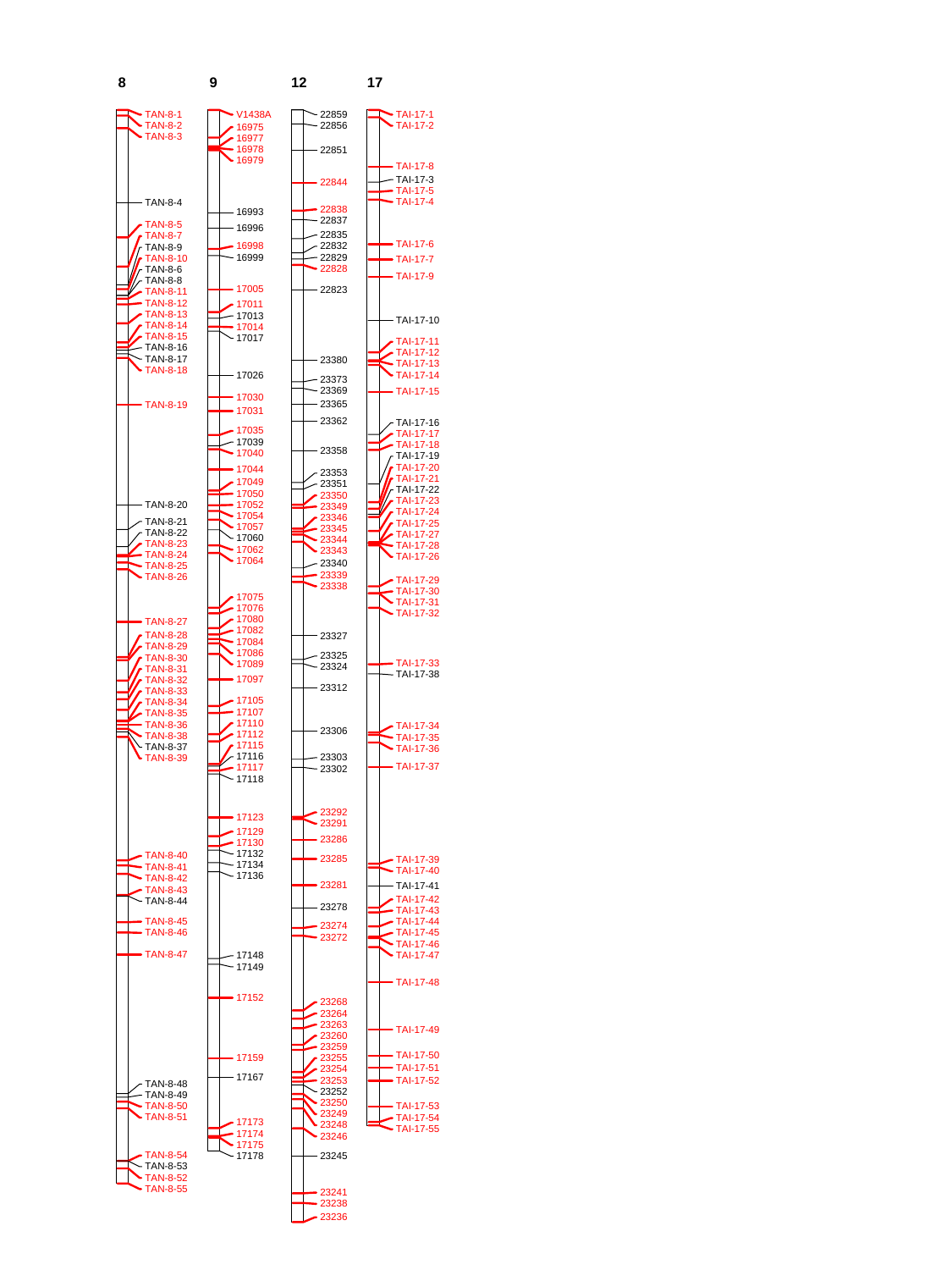

Supplement: Additional file 3: Figure S1. — Position of the amplicons selected in the four LD study regions for SNP discovery by sequencing. For amplicons in red, at least one SNP was successfully genotyped with Illumina® VeraCode®. (PPTX 89 kb) [file 12870_2016_754_MOESM3_ESM.pptx]

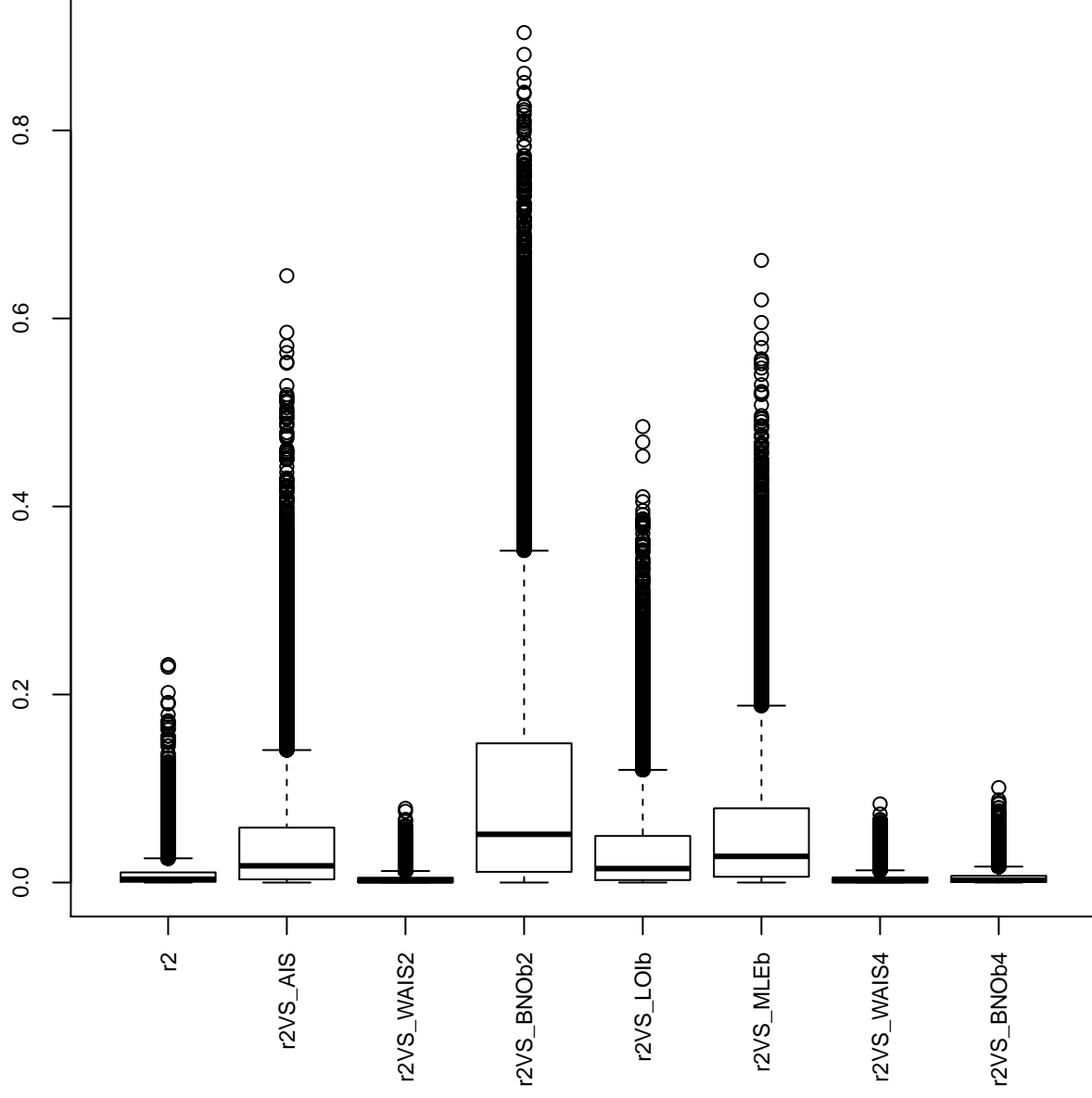

Supplement: Additional file 6: Figure S2. — Comparison of the distribution of the r 2 VS LD values between unlinked SNPs of the four genomic regions, corrected by both structure and one of seven kinship matrices, for the whole association panel. The boxplot for r 2 is the uncorrected reference. (PDF 76 kb) [file 12870_2016_754_MOESM6_ESM.pdf]

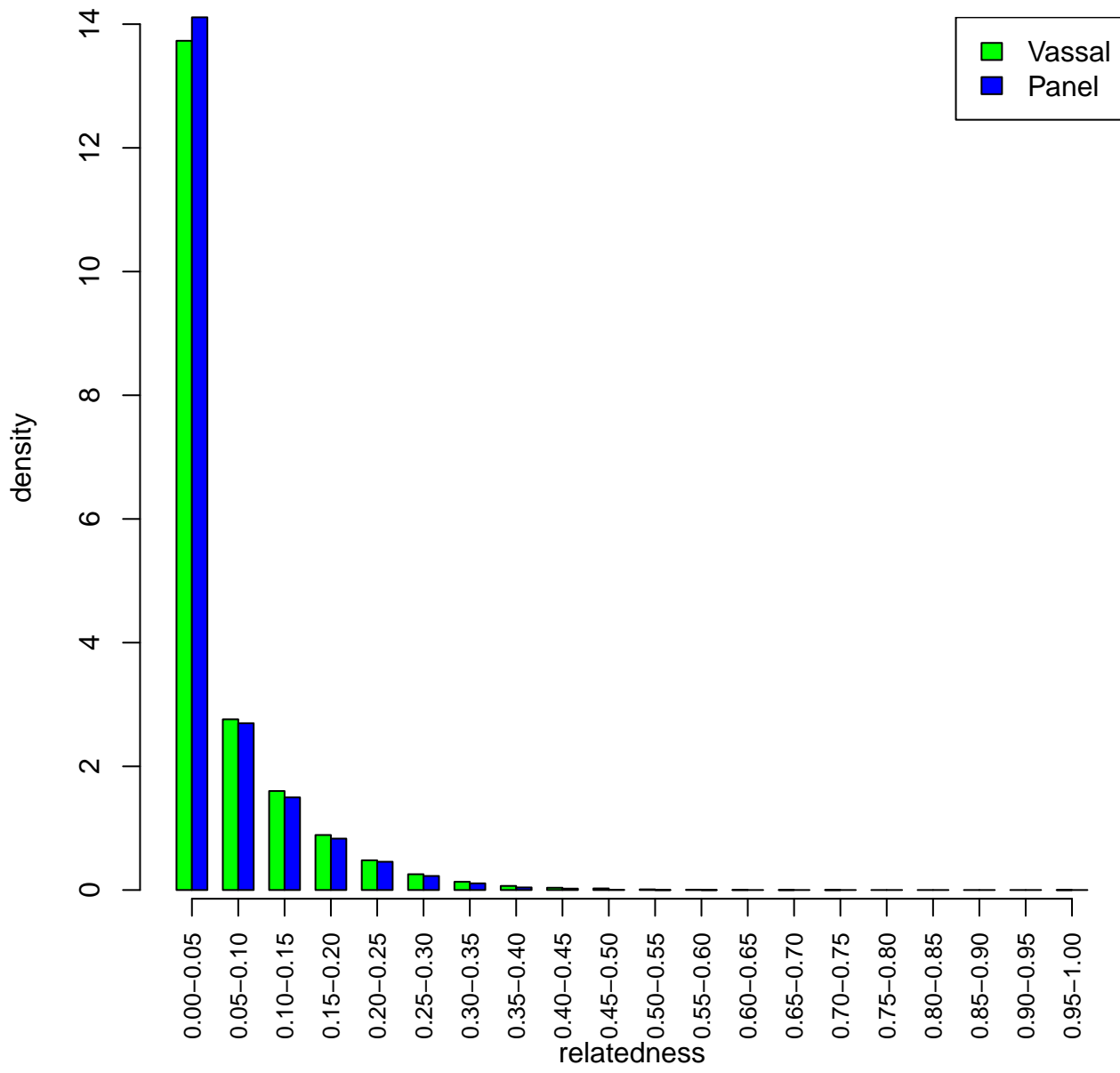

Supplement: Additional file 9: Figure S3. — Histogram of relatedness in the Vassal collection vs in the association panel, based on 20 SSRs. (PDF 4 kb) [file 12870_2016_754_MOESM9_ESM.pdf]

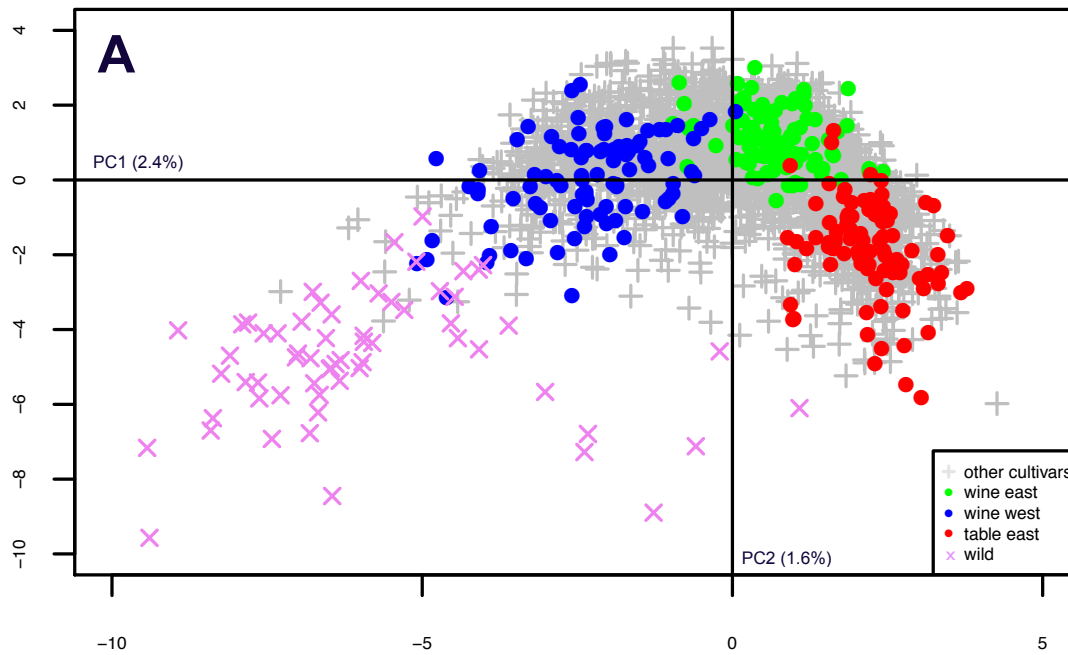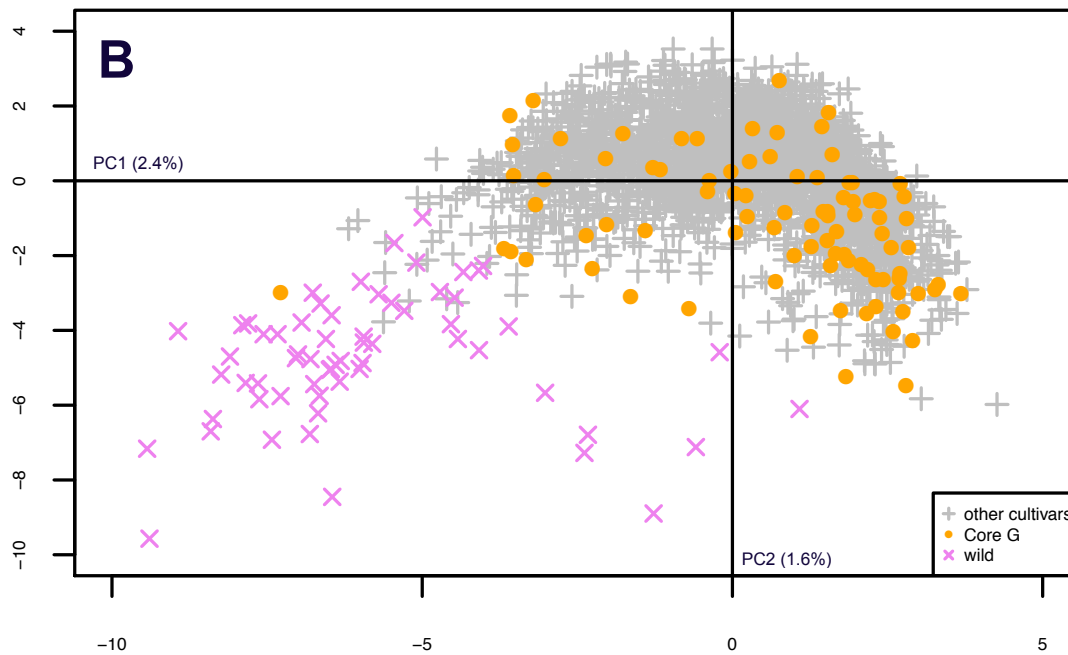

Supplement: Additional file 10: Figure S4. — PCA analysis of the Vassal collection and wild panel, based on SSR data. Relative position of: A. The three subgroups of the association panel. B. The genetic core collection. (PDF 254 kb) [file 12870_2016_754_MOESM10_ESM.pdf]

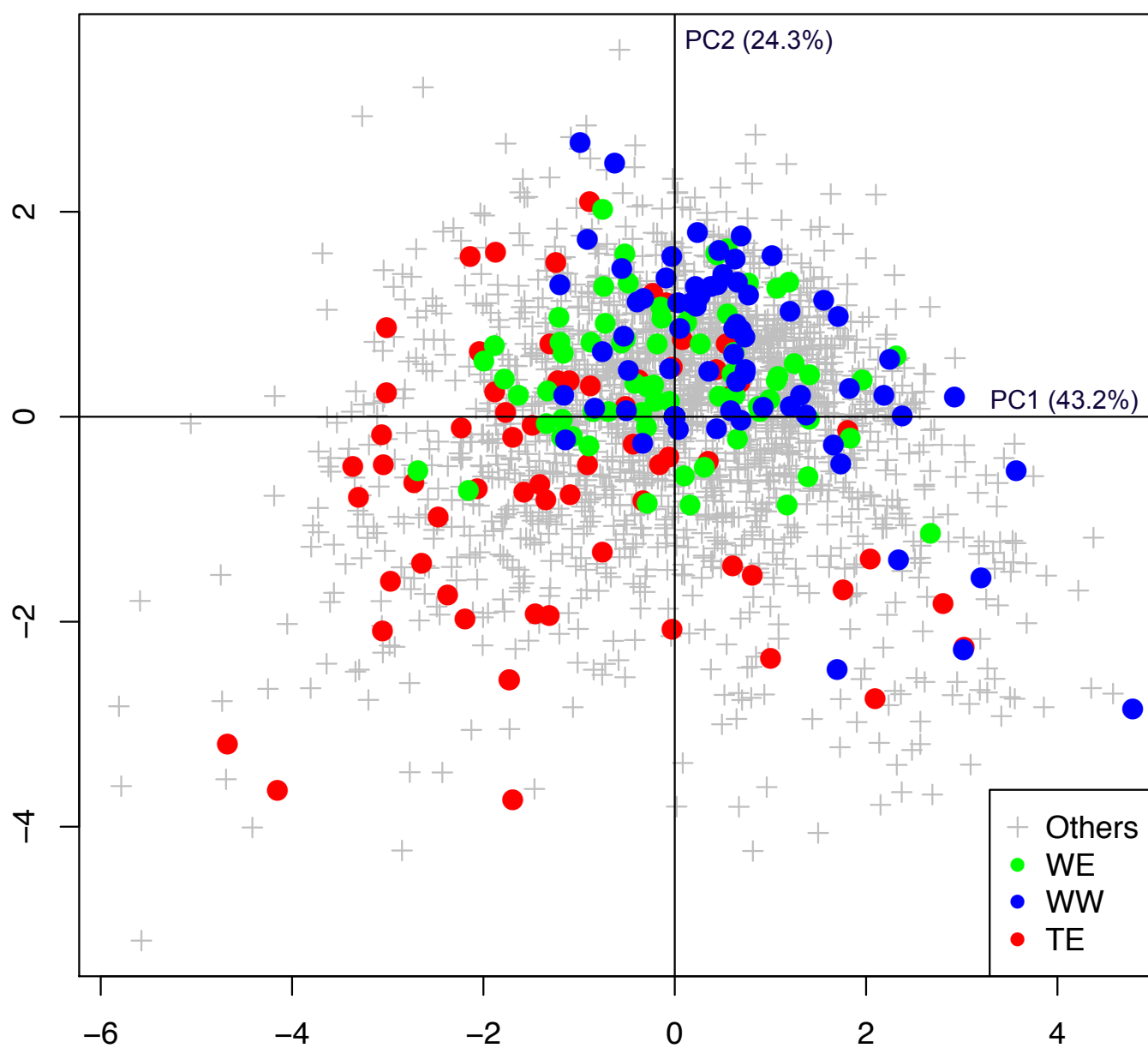

Supplement: Additional file 12: Figure S5. — PCA analysis for comparing the association panel with the whole Vassal collection, based on five quantitative traits. (PDF 86 kb) [file 12870_2016_754_MOESM12_ESM.pdf]

WE vs. TE

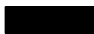

WW vs. TE

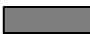

WW vs. WE

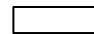

Fst value

0.08

0.06

0.04

0.02

SNPs\_Ch08

SNPs\_Ch09

SNPs\_Ch12

SNPs\_Ch17

SNPs\_distr

SSRn

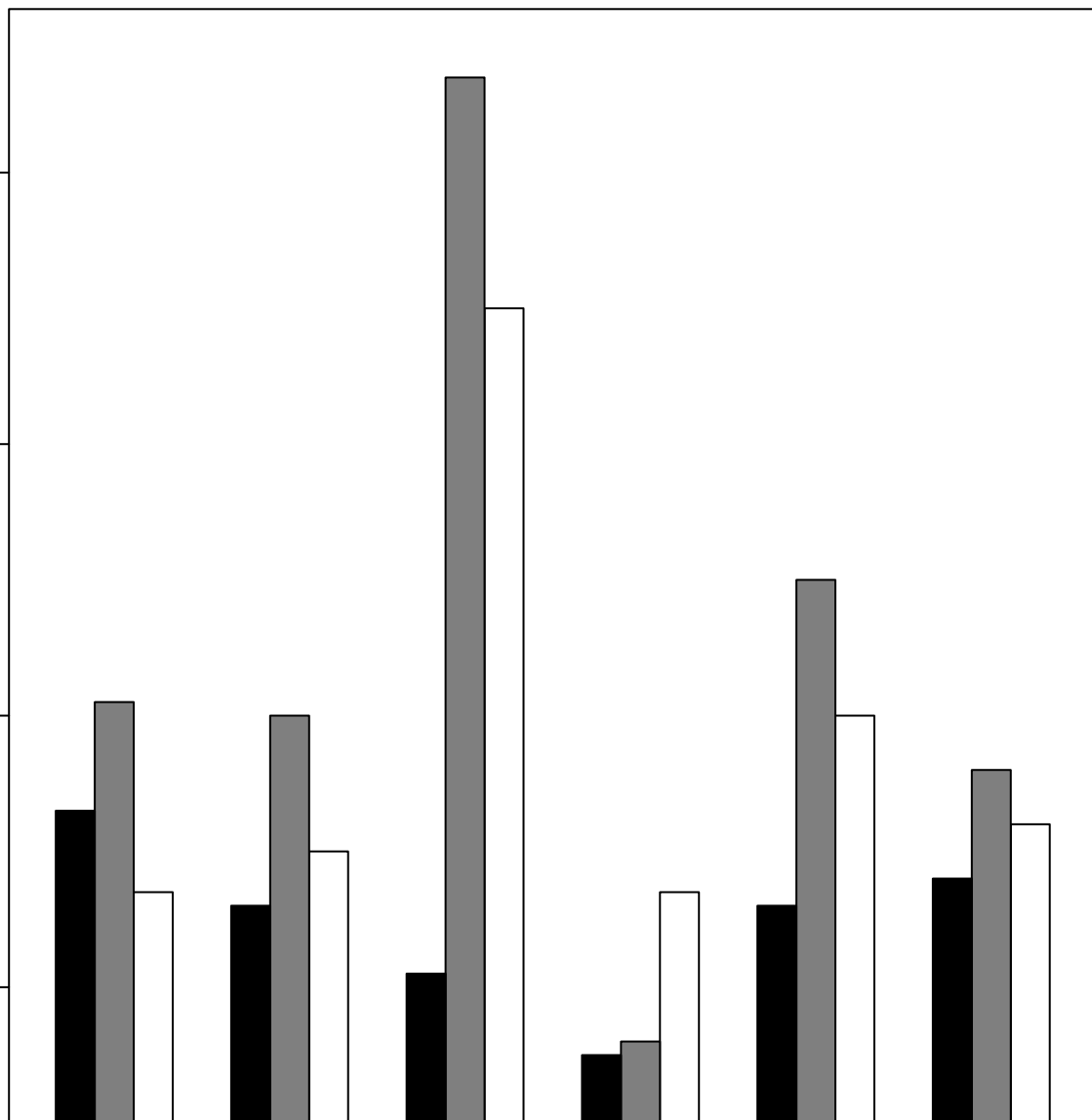

Supplement: Additional file 14: Figure S6. — Pairwise genetic differentiation (F st) amongst subgroups (based on SNPs or SSRs). All F st values were significantly different from zero according to the test implemented in GenAlEx, based on 1000 permutations. WW: wine West, WE: wine East, TE: table East. (PDF 8 kb) [file 12870_2016_754_MOESM14_ESM.pdf]

# Chromosome 8

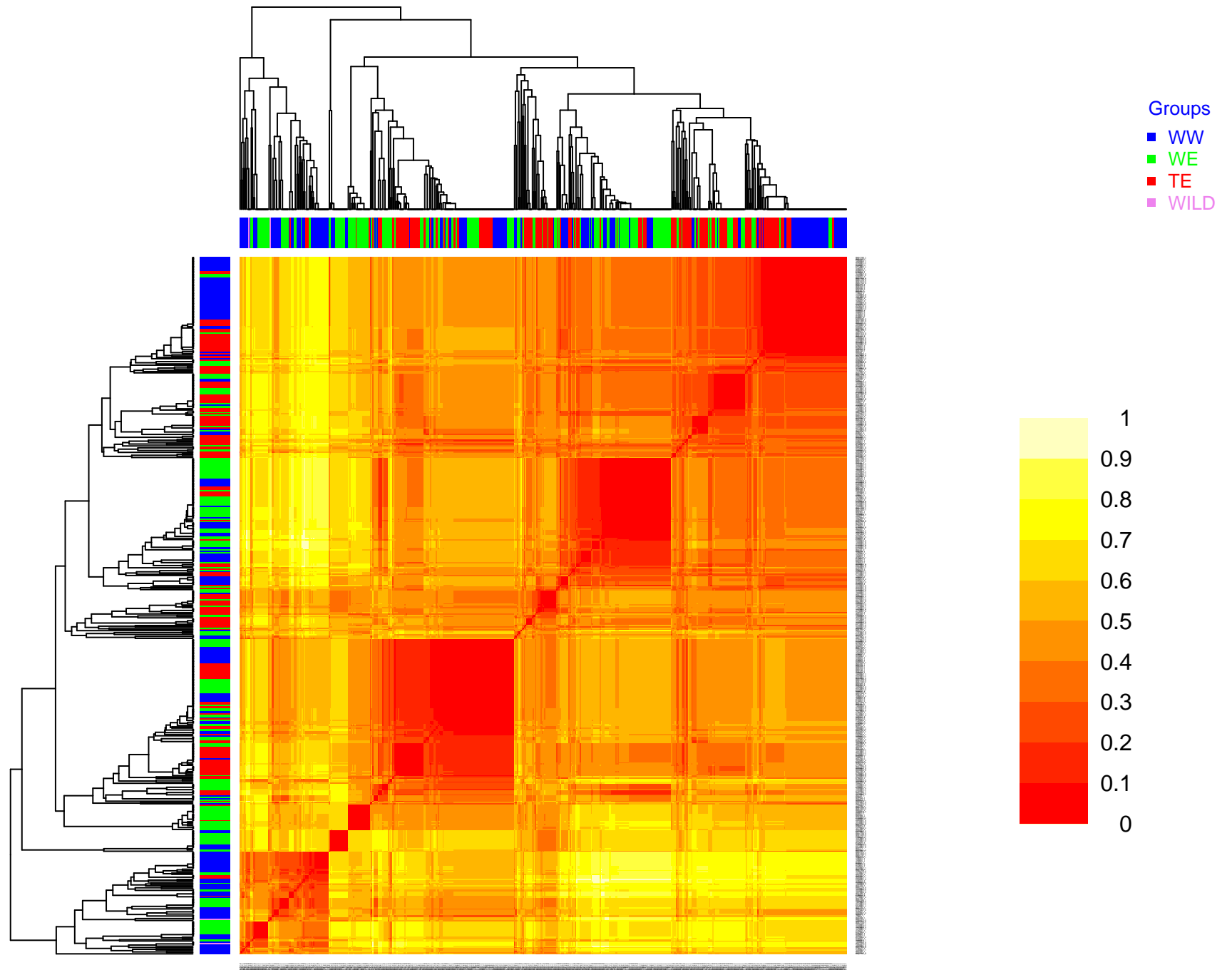

# Chromosome 9

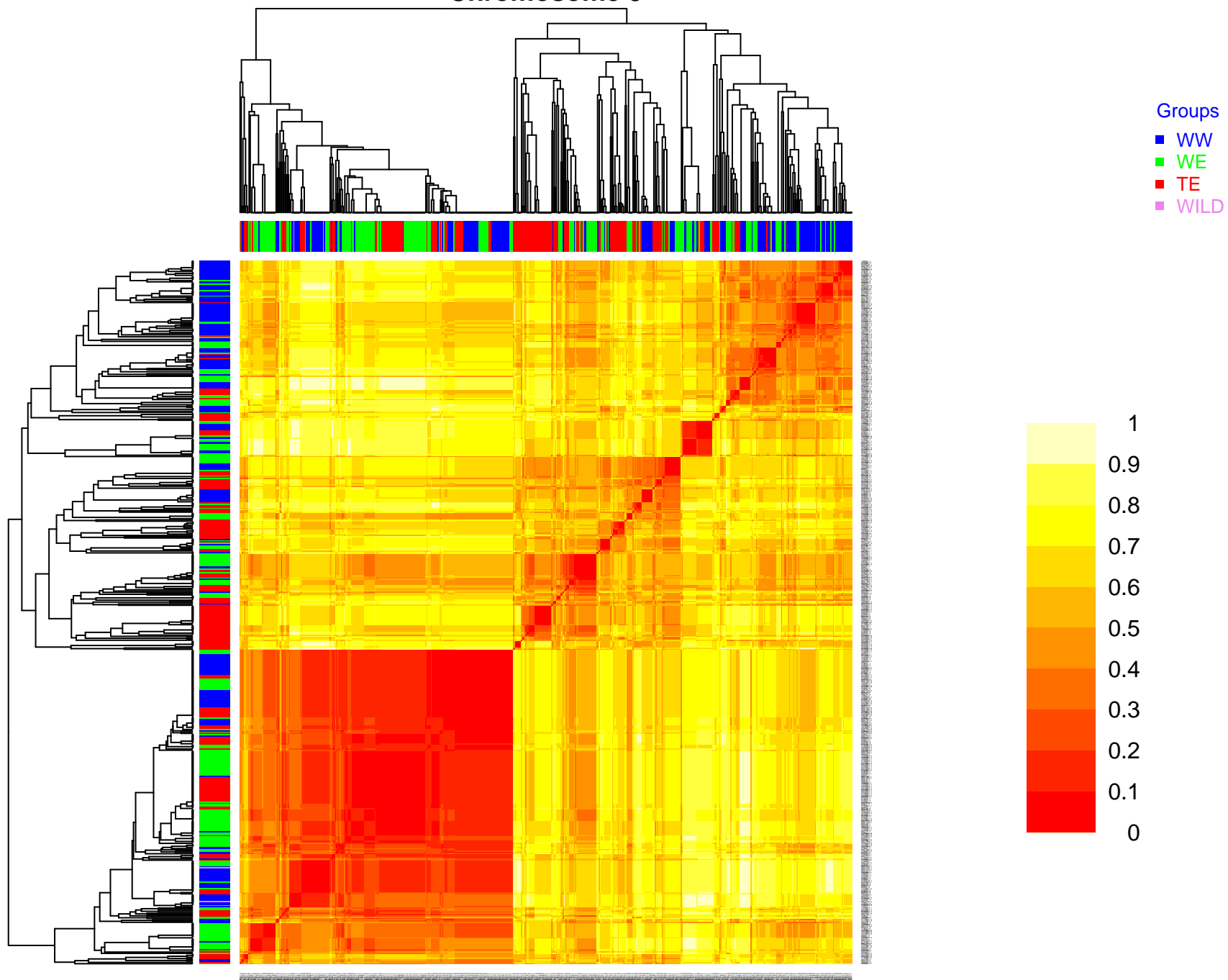

## Chromosome 12

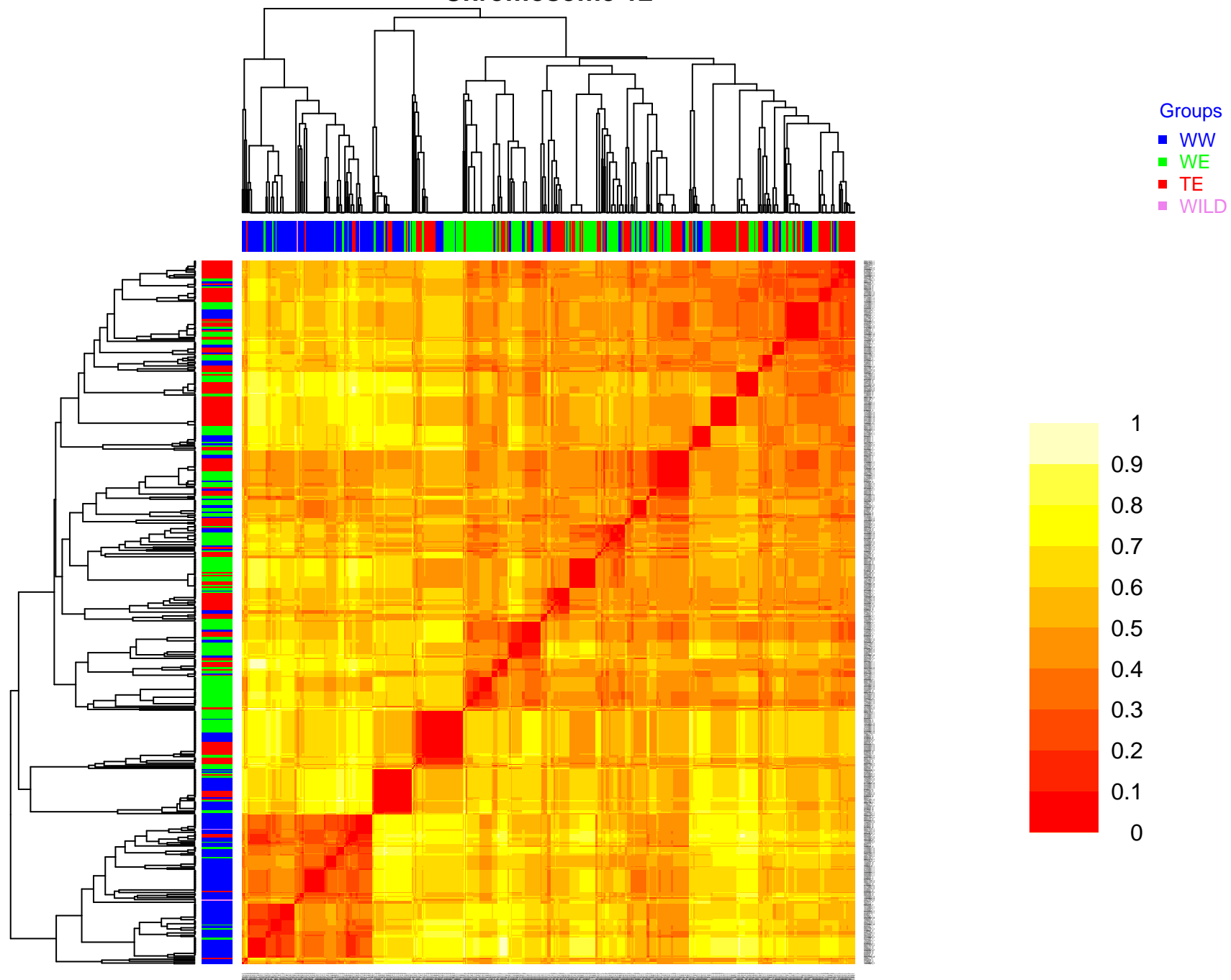

Chromosome 17

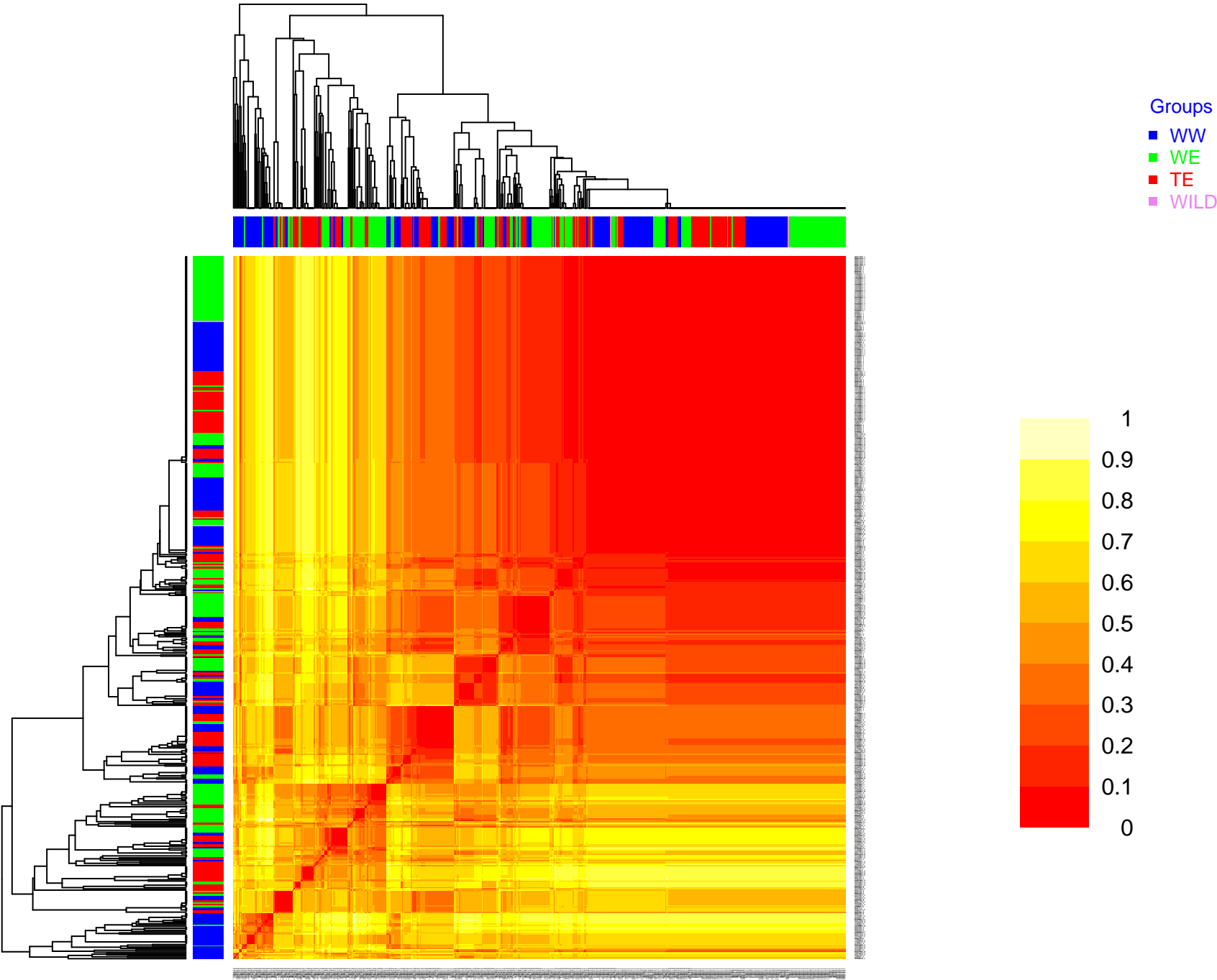

Supplement: Additional file 17: Figure S9. — IBS clustering of reconstructed haplotypes in the four genomic regions. (PDF 4111 kb) [file 12870_2016_754_MOESM17_ESM.pdf]

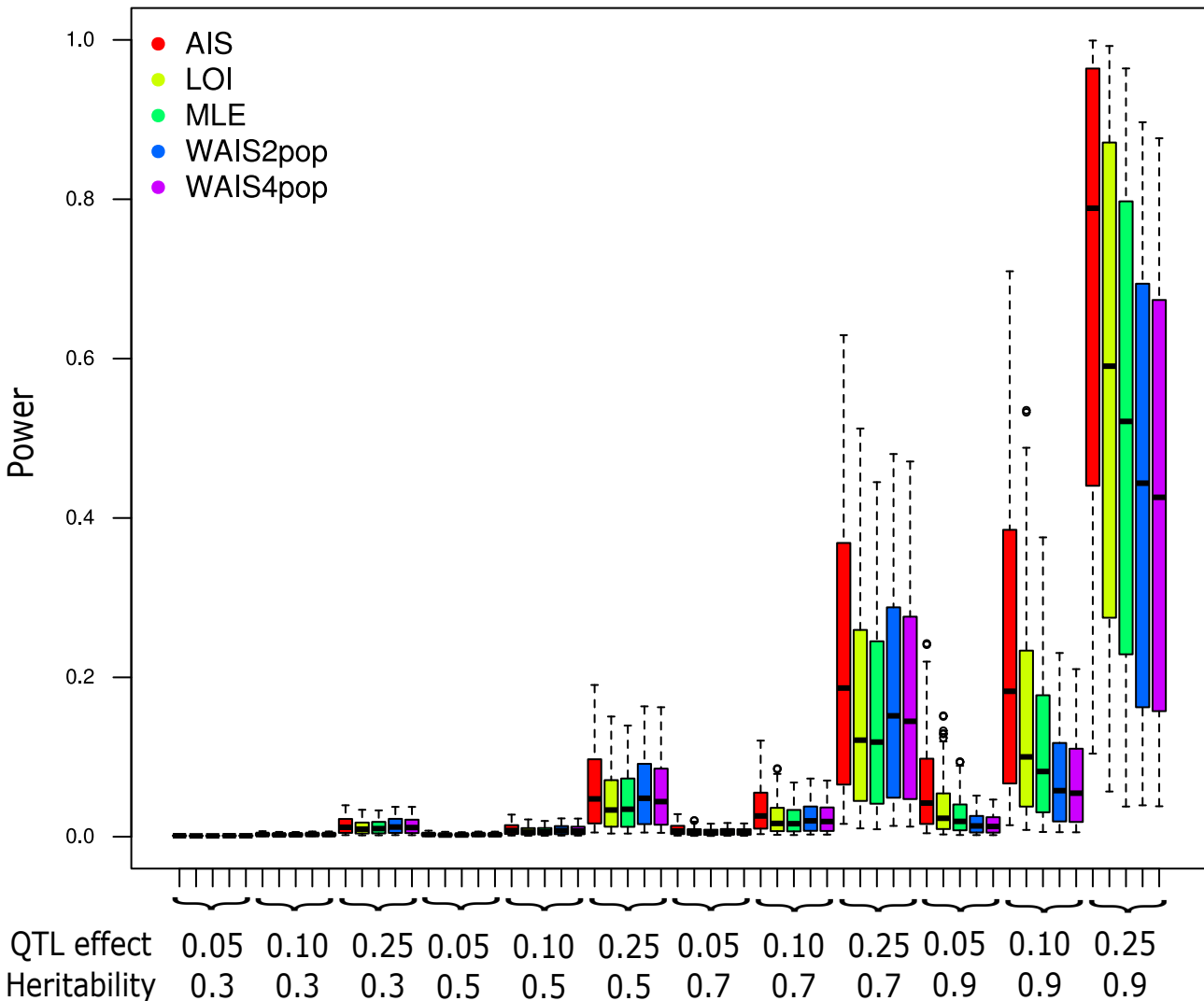

Supplement: Additional file 18: Figure S10. — Variation of power distribution at 372 SNPs using five different kinship estimators. We used a 5 % family wise error rate and various heritability and QTL effect values. (PDF 19 kb) [file 12870_2016_754_MOESM18_ESM.pdf]

QTL effect

0.05

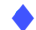

0.1

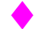

0.25

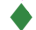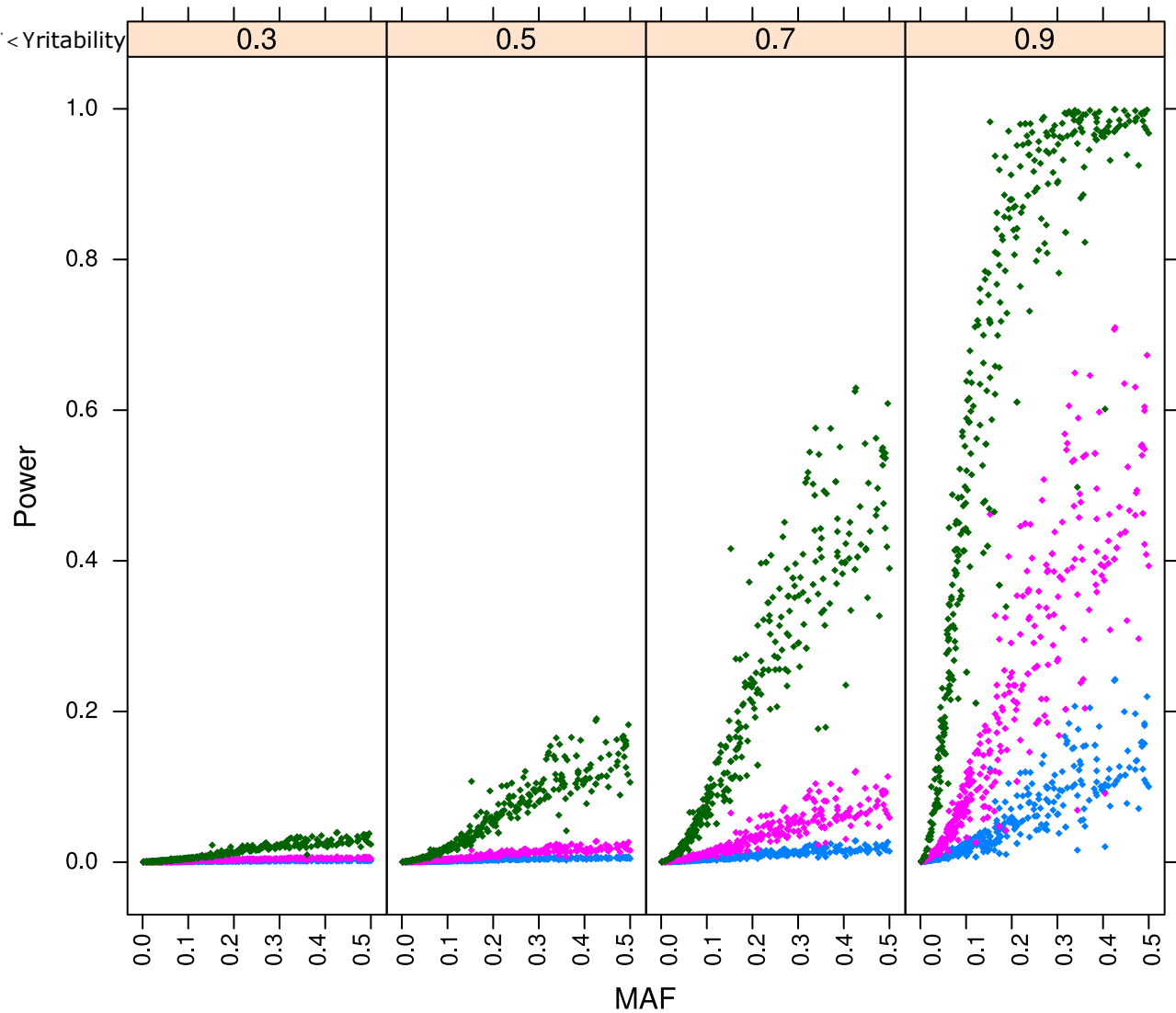

Supplement: Additional file 19: Figure S11. — Variation of power according to minor allele frequency (MAF), with a 5 % family wise error rate and using AIS kinship, for different levels of heritability and QTL effect. (PDF 236 kb) [file 12870_2016_754_MOESM19_ESM.pdf]
