# Supplementary material for: Genetic diversity, linkage disequilibrium and power of a large grapevine (Vitis vinifera L) diversity panel newly designed for association studies
Source: BMC Plant Biol. 2016 Mar 22;16:74. doi: 10.1186/s12870-016-0754-z (PMC4802926; doi:10.1186/s12870-016-0754-z)
Supplement: Additional file 15: Figure S7. — Mean local genotypic LD in a 300 Kb-sliding window along the genomic regions on chromosomes 8, 9 and 12 in each subgroup of the association panel (WE, WW and TE) and the wild panel. Only mean LD values based on at least ten marker pairs are plotted. Vertical lines on the x-axis indicate SNP positions. (PDF 19 kb) [file 12870_2016_754_MOESM15_ESM.pdf]

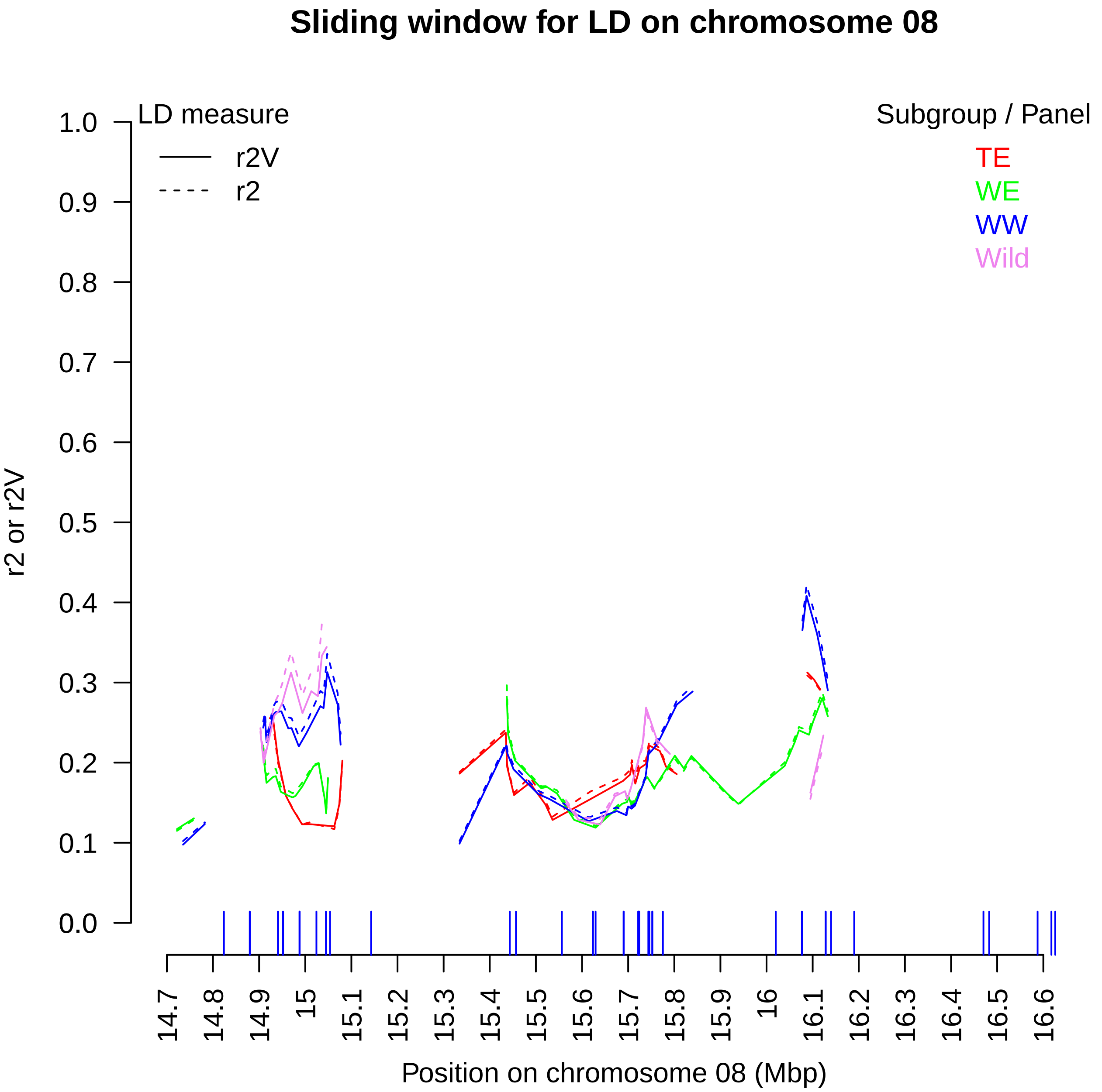

Sliding window for LD on chromosome 08
LD measure
r2V
r2
Subgroup / Panel
TE
WE
WW
Wild
r2 or r2V
1.0
0.9
0.8
0.7
0.6
0.5
0.4
0.3
0.2
0.1
0.0
14.7
14.8
14.9
15
15.1
15.2
15.3
15.4
15.5
15.6
15.7
15.8
15.9
16
16.1
16.2
16.3
16.4
16.5
16.6
Position on chromosome 08 (Mbp)

Sliding window for LD on chromosome 09

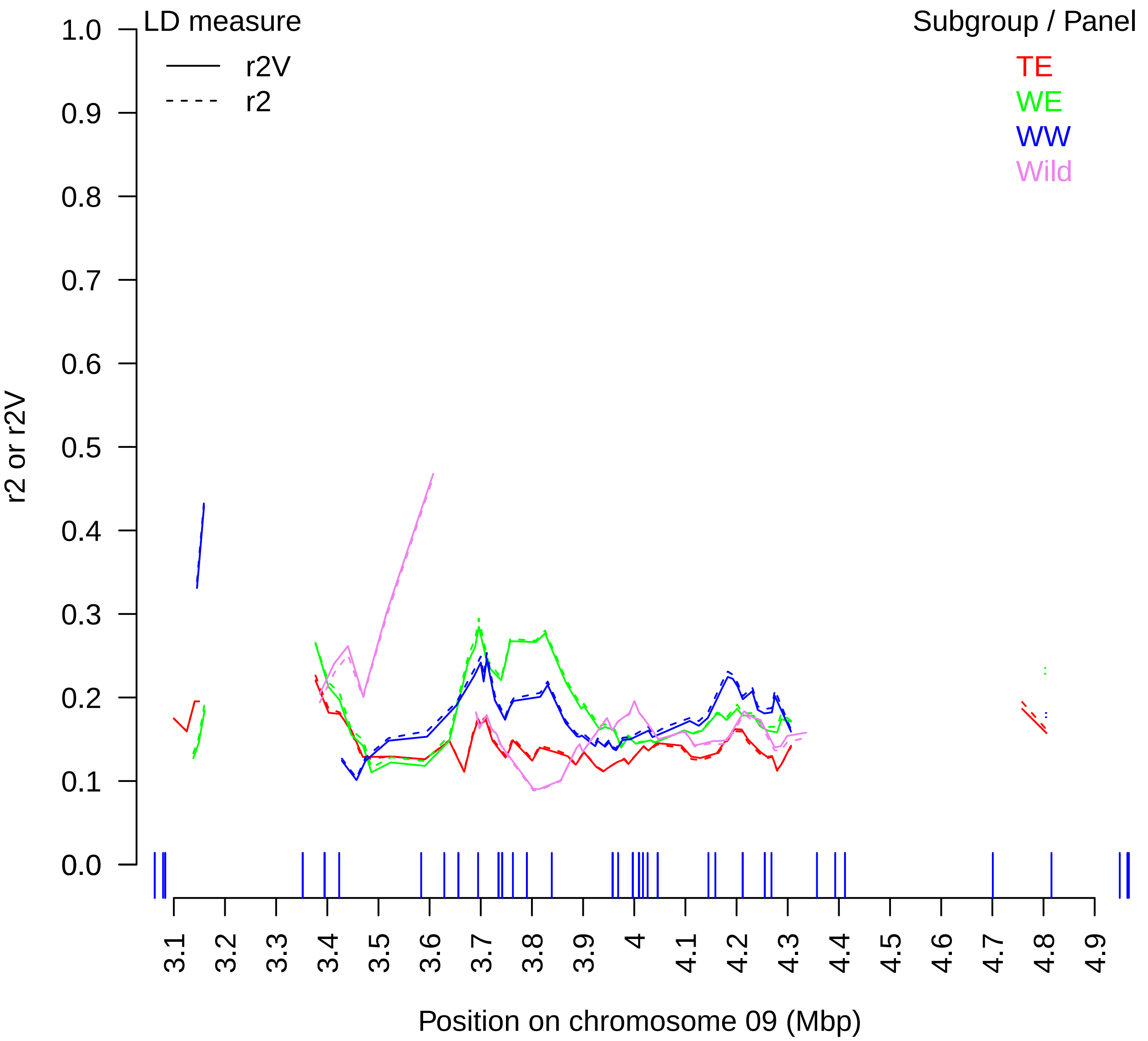

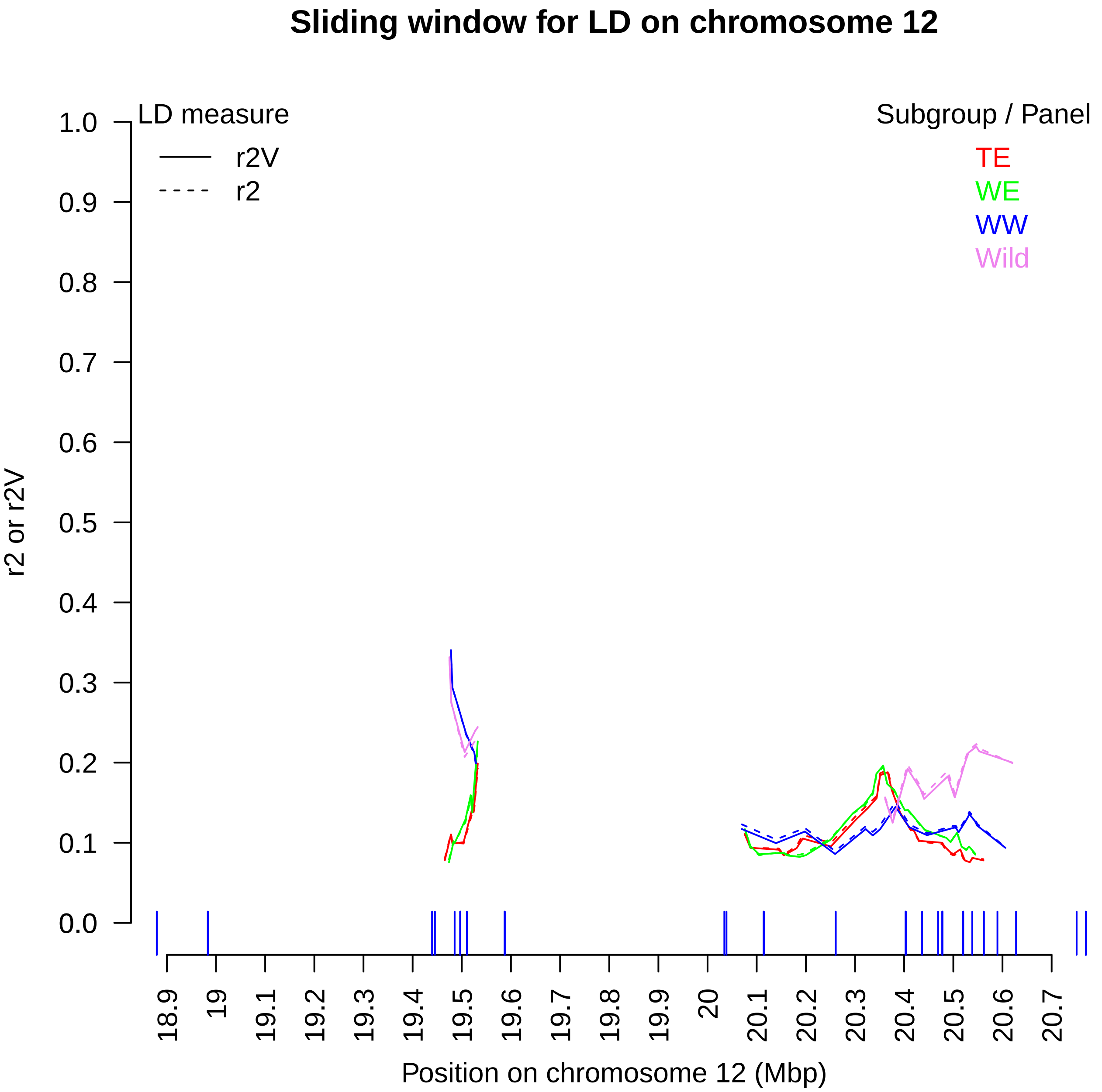

Sliding window for LD on chromosome 12
LD measure
r2V
r2
Subgroup / Panel
TE
WE
WW
Wild
r2 or r2V
1.0
0.9
0.8
0.7
0.6
0.5
0.4
0.3
0.2
0.1
0.0
18.9
19
19.1
19.2
19.3
19.4
19.5
19.6
19.7
19.8
19.9
20
20.1
20.2
20.3
20.4
20.5
20.6
20.7
Position on chromosome 12 (Mbp)
