# Supplementary material for: Genetic diversity, linkage disequilibrium and power of a large grapevine (Vitis vinifera L) diversity panel newly designed for association studies
Source: BMC Plant Biol. 2016 Mar 22;16:74. doi: 10.1186/s12870-016-0754-z (PMC4802926; doi:10.1186/s12870-016-0754-z)
Supplement: Additional file 16: Figure S8. — Mean local Nei’s diversity (A) and F st (B) in a 300 Kb-sliding window along the genomic regions on chromosomes 8, 9, 12 and 17 for each subgroup of the association panel (WE, WW and TE) and the wild panel. Only mean values based on at least five markers are plotted. Vertical lines on the x-axis indicate SNP positions. (PDF 39 kb) [file 12870_2016_754_MOESM16_ESM.pdf]

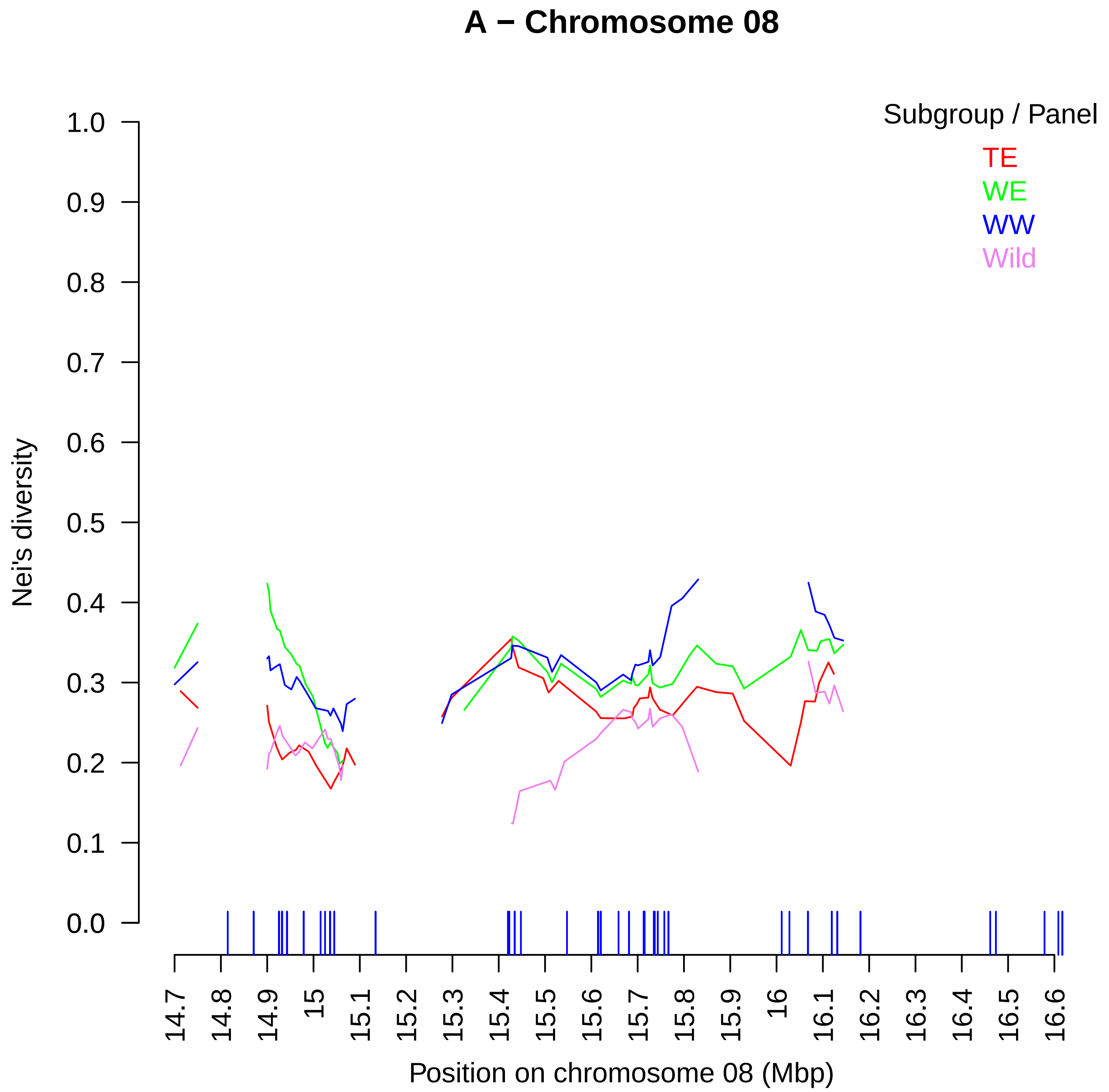

A − Chromosome 08
Subgroup / Panel
TE
WE
WW
Wild
Nei's diversity
1.0
0.9
0.8
0.7
0.6
0.5
0.4
0.3
0.2
0.1
0.0
14.7
14.8
14.9
15
15.1
15.2
15.3
15.4
15.5
15.6
15.7
15.8
15.9
16
16.1
16.2
16.3
16.4
16.5
16.6
Position on chromosome 08 (Mbp)

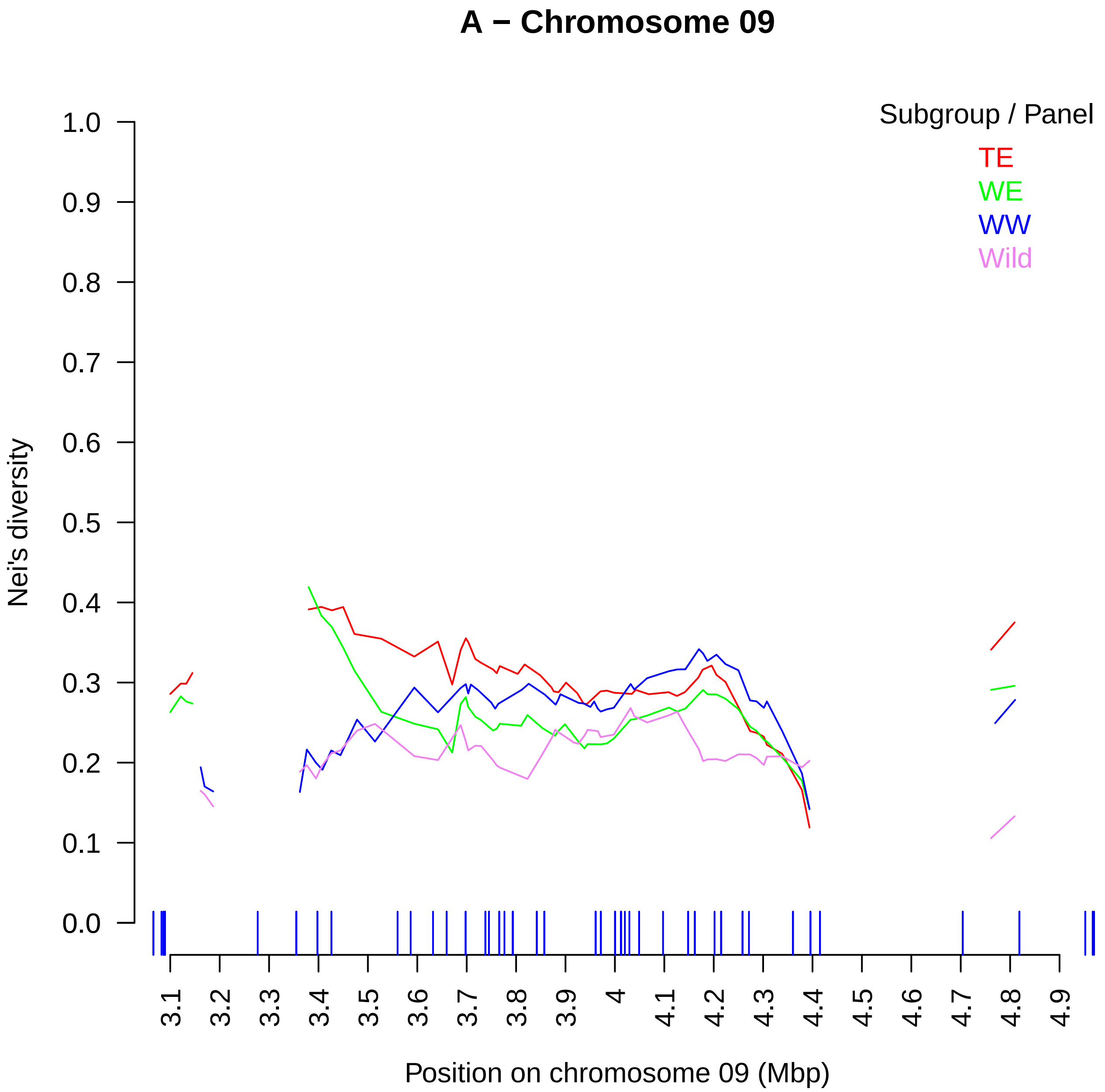
A − Chromosome 09
Subgroup / Panel
TE
WE
WW
Wild
Nei's diversity
1.0
0.9
0.8
0.7
0.6
0.5
0.4
0.3
0.2
0.1
0.0
3.1
3.2
3.3
3.4
3.5
3.6
3.7
3.8
3.9
4
4.1
4.2
4.3
4.4
4.5
4.6
4.7
4.8
4.9
Position on chromosome 09 (Mbp)

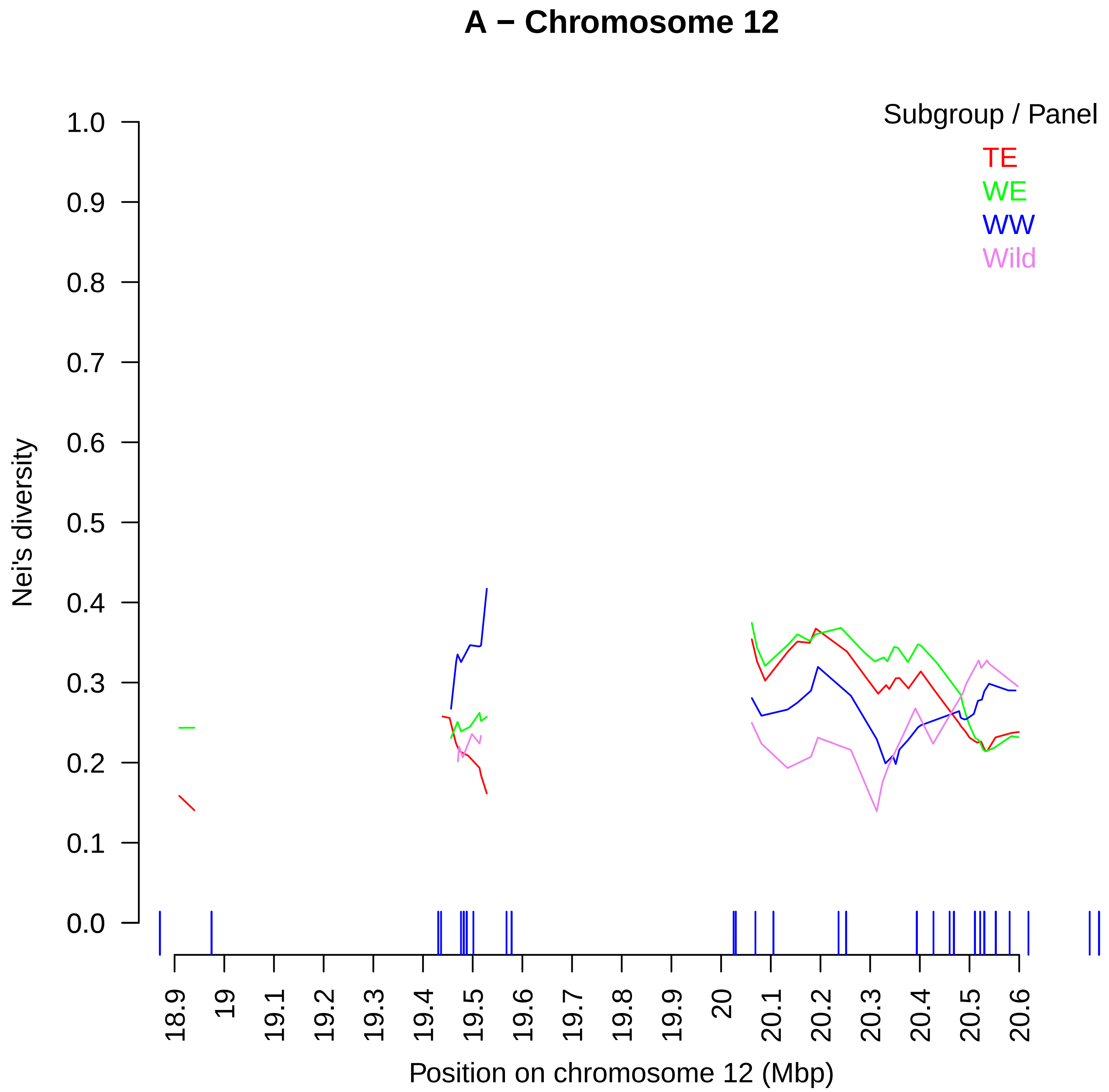

A − Chromosome 12
Subgroup / Panel
TE
WE
WW
Wild
Nei's diversity
1.0
0.9
0.8
0.7
0.6
0.5
0.4
0.3
0.2
0.1
0.0
18.9
19
19.1
19.2
19.3
19.4
19.5
19.6
19.7
19.8
19.9
20
20.1
20.2
20.3
20.4
20.5
20.6
Position on chromosome 12 (Mbp)

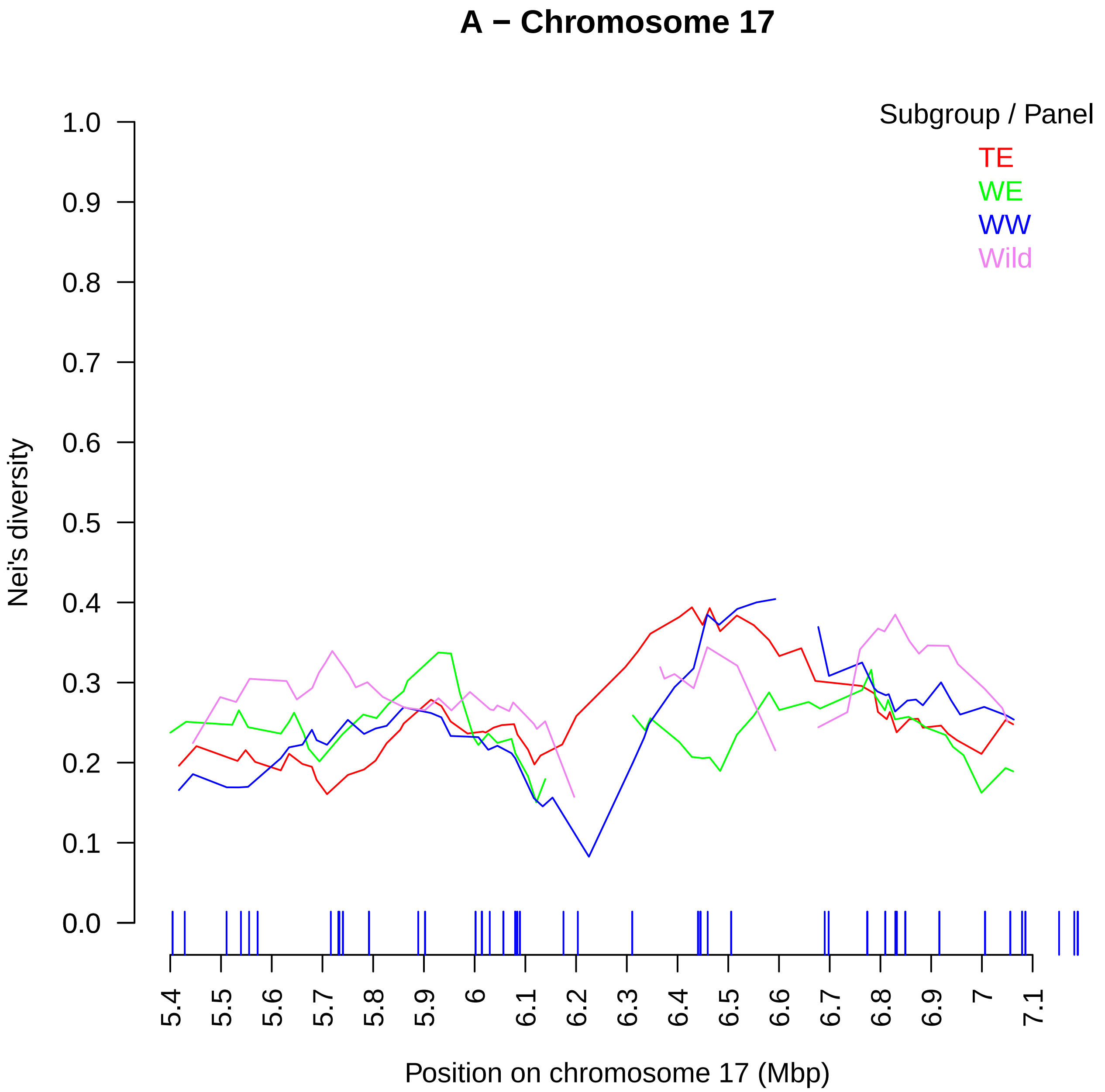

A − Chromosome 17
Subgroup / Panel
TE
WE
WW
Wild
Nei's diversity
1.0
0.9
0.8
0.7
0.6
0.5
0.4
0.3
0.2
0.1
0.0
5.4
5.5
5.6
5.7
5.8
5.9
6
6.1
6.2
6.3
6.4
6.5
6.6
6.7
6.8
6.9
7
7.1
Position on chromosome 17 (Mbp)

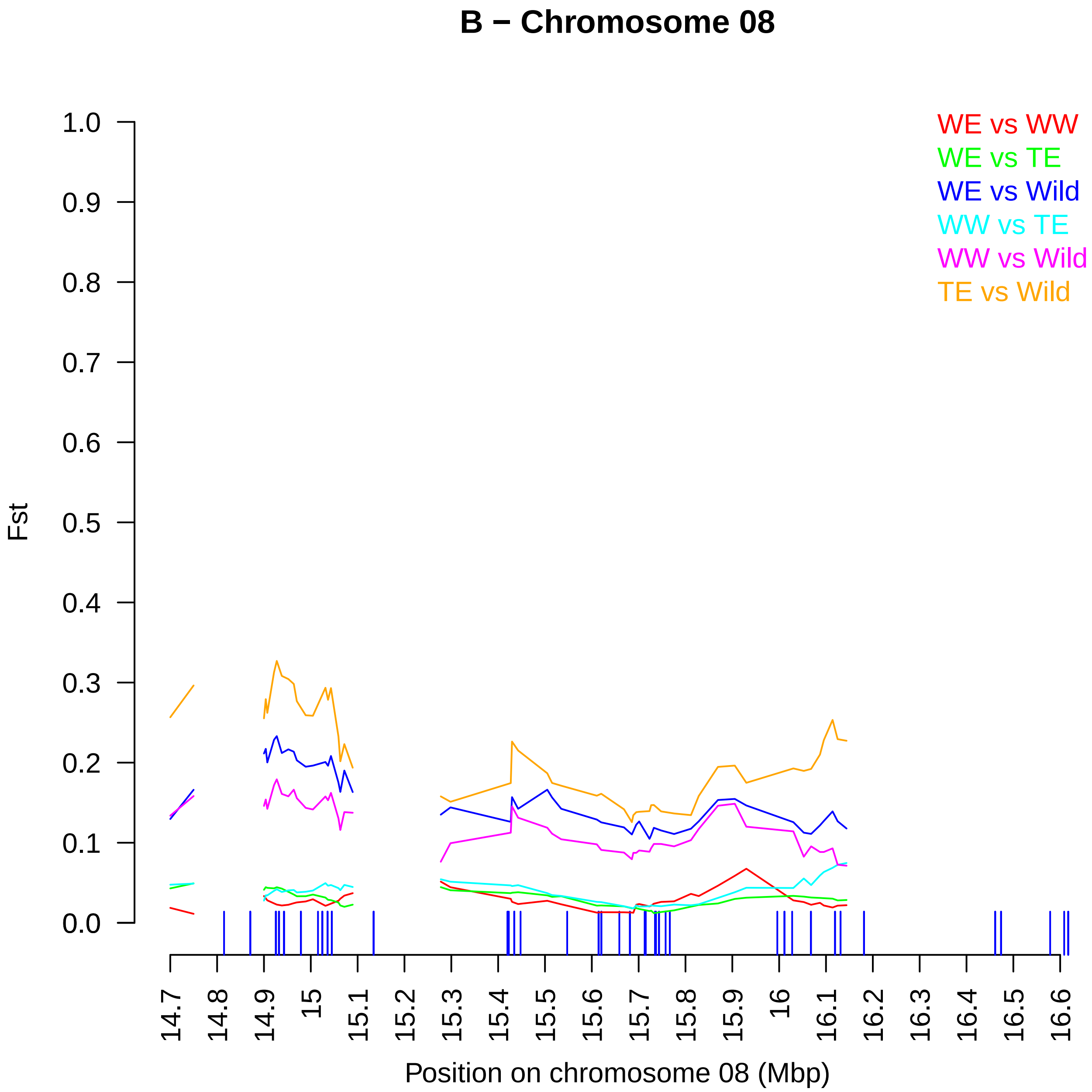
B − Chromosome 08
WE vs WW
WE vs TE
WE vs Wild
WW vs TE
WW vs Wild
TE vs Wild
Fst
1.0
0.9
0.8
0.7
0.6
0.5
0.4
0.3
0.2
0.1
0.0
14.7
14.8
14.9
15
15.1
15.2
15.3
15.4
15.5
15.6
15.7
15.8
15.9
16
16.1
16.2
16.3
16.4
16.5
16.6
Position on chromosome 08 (Mbp)

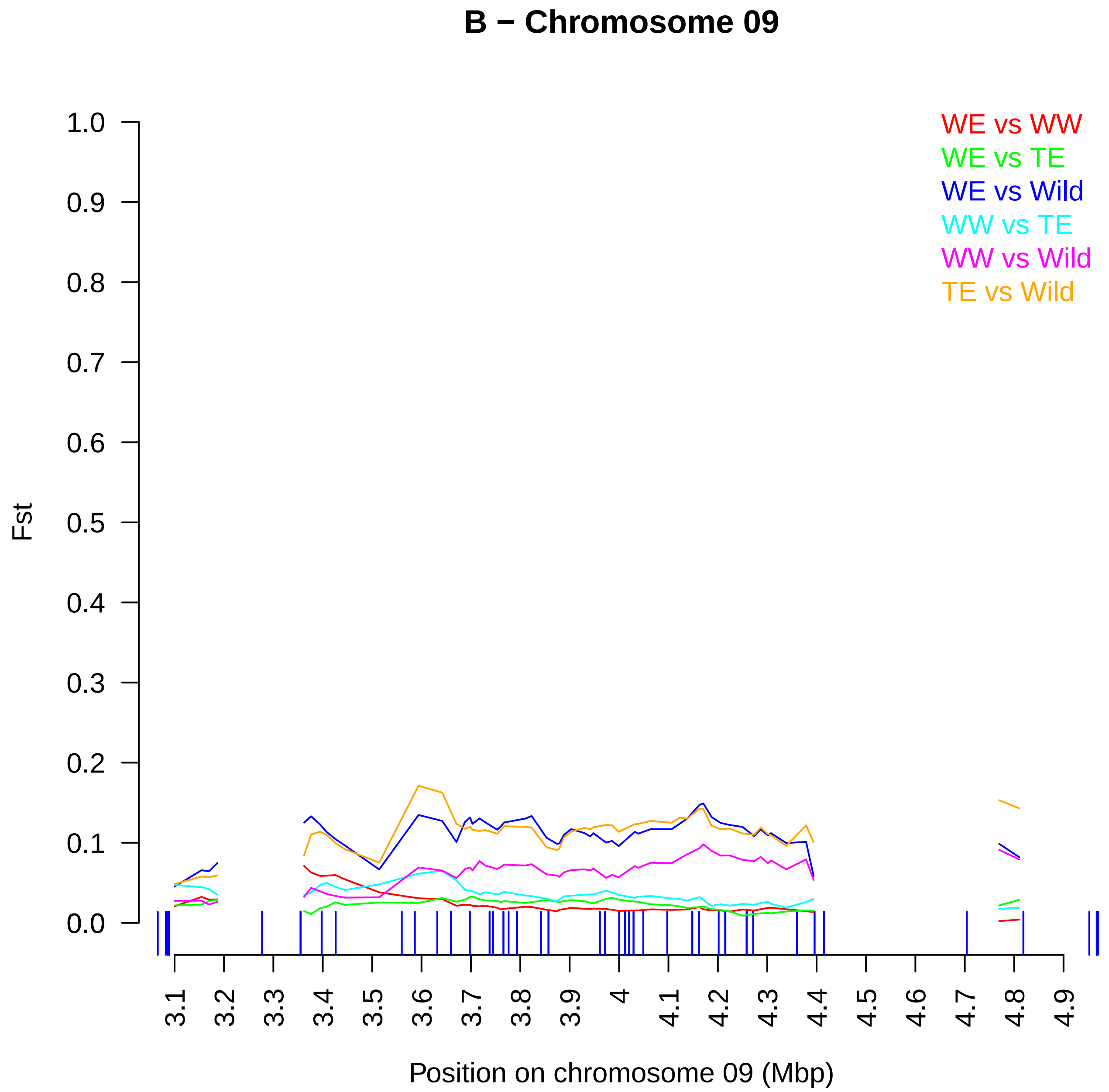
B − Chromosome 09
Fst
1.0
0.9
0.8
0.7
0.6
0.5
0.4
0.3
0.2
0.1
0.0
WE vs WW
WE vs TE
WE vs Wild
WW vs TE
WW vs Wild
TE vs Wild
3.1
3.2
3.3
3.4
3.5
3.6
3.7
3.8
3.9
4
4.1
4.2
4.3
4.4
4.5
4.6
4.7
4.8
4.9
Position on chromosome 09 (Mbp)

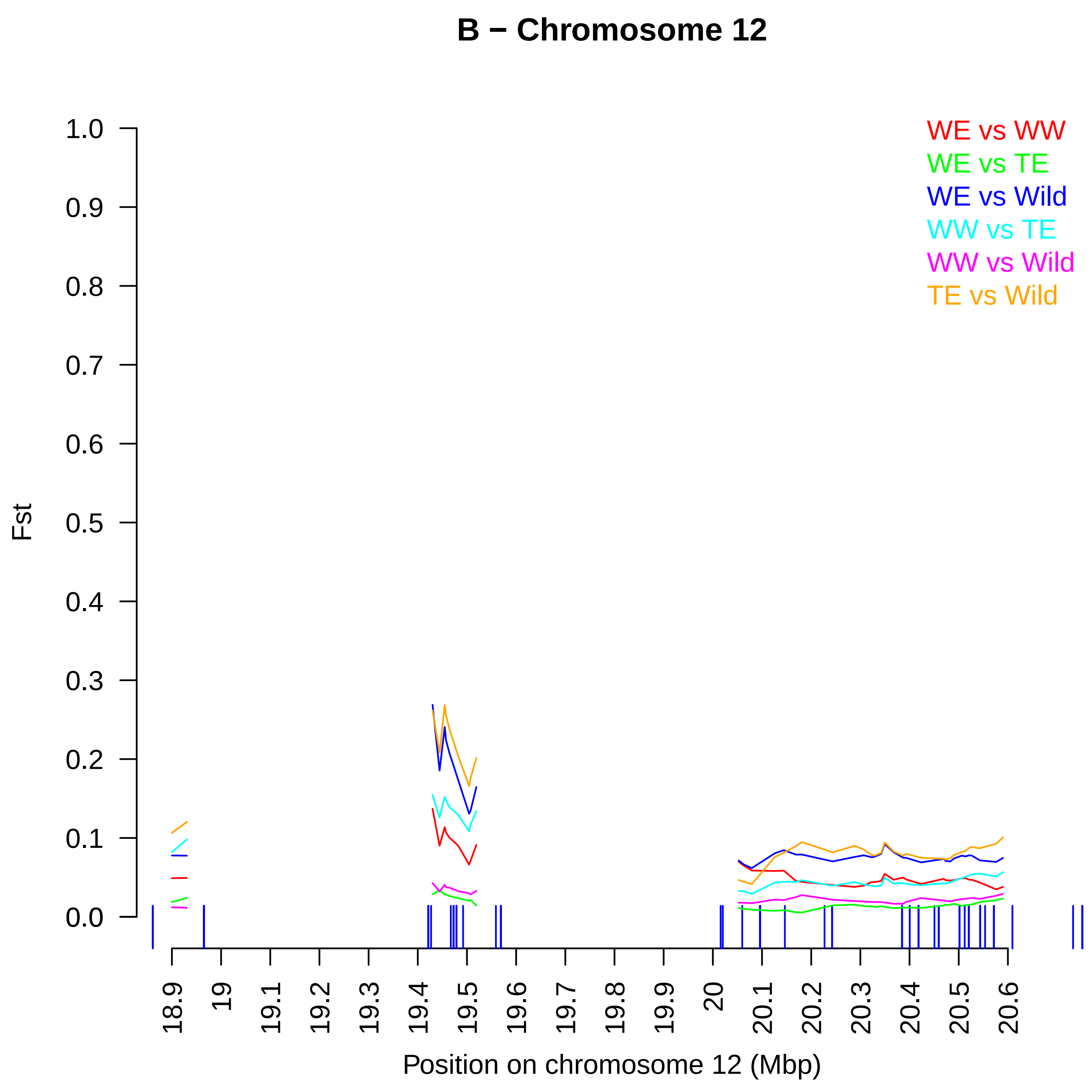
B − Chromosome 12
WE vs WW
WE vs TE
WE vs Wild
WW vs TE
WW vs Wild
TE vs Wild
Fst
1.0
0.9
0.8
0.7
0.6
0.5
0.4
0.3
0.2
0.1
0.0
18.9
19
19.1
19.2
19.3
19.4
19.5
19.6
19.7
19.8
19.9
20
20.1
20.2
20.3
20.4
20.5
20.6
Position on chromosome 12 (Mbp)

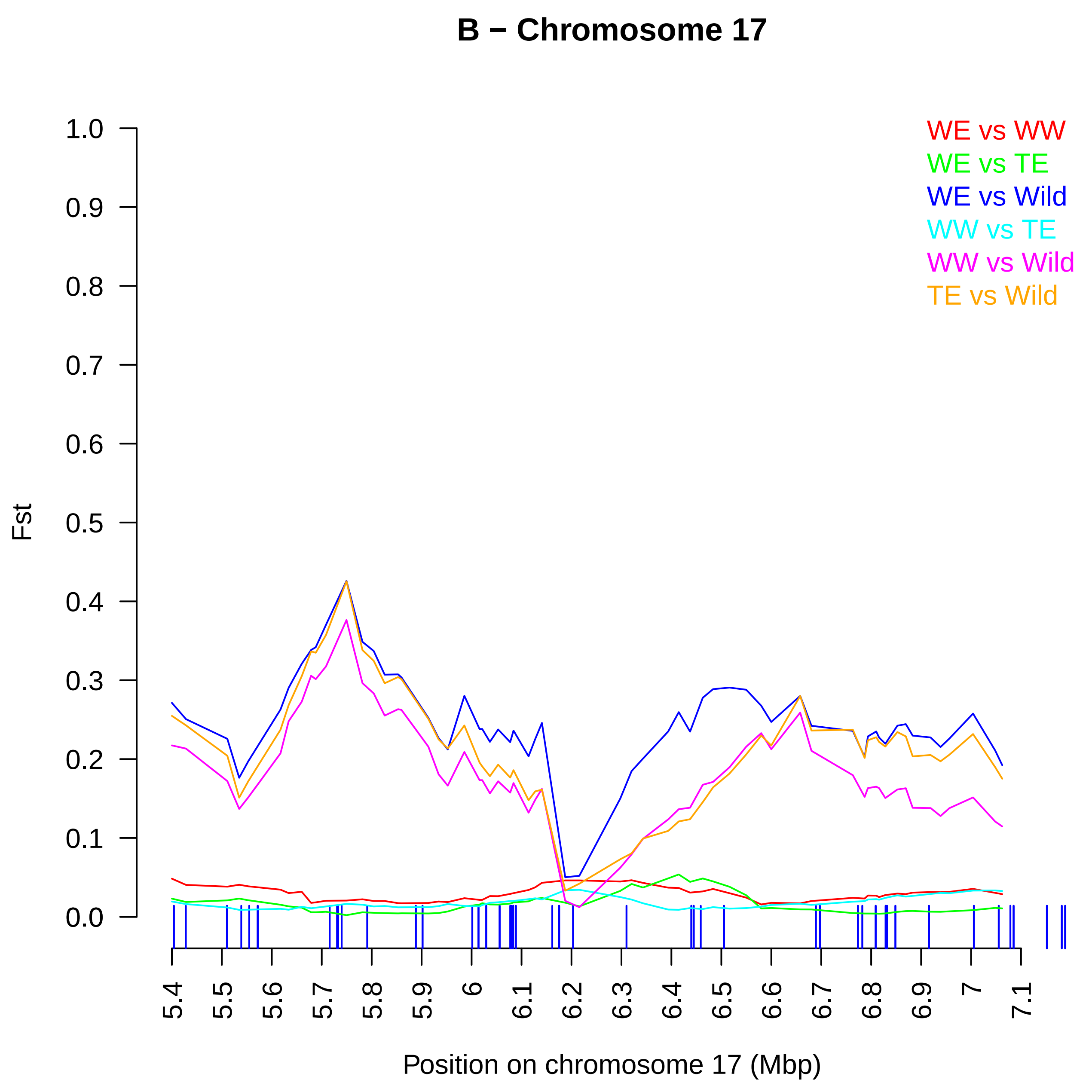
B − Chromosome 17
Fst
1.0
0.9
0.8
0.7
0.6
0.5
0.4
0.3
0.2
0.1
0.0
WE vs WW
WE vs TE
WE vs Wild
WW vs TE
WW vs Wild
TE vs Wild
5.4
5.5
5.6
5.7
5.8
5.9
6
6.1
6.2
6.3
6.4
6.5
6.6
6.7
6.8
6.9
7
7.1
Position on chromosome 17 (Mbp)
